# Structural Features of a Tiny Viral Protein, ORF7b of SARS-CoV-2

**DOI:** 10.3390/ijms27136022

**Published:** 2026-07-04

**Authors:** Giovanni Colonna

**Affiliations:** Medical Informatics Unit, Azienda Ospedaliera Universitaria Luigi Vanvitelli, Università degli Studi della Campania, 80138 Naples, Italy; giovanni.colonna@unicampania.it

**Keywords:** ORF7b of SARS-CoV-2, COVID-19, 3D structure, molecular dynamics, dipole vector, electrostatic properties, protein flexibility, SARS protein interactions, RIN analysis, peripheral membrane protein

## Abstract

Accessory proteins of SARS-CoV-2 play crucial roles in viral pathogenesis, yet their structural properties remain elusive. ORF7b, a small accessory protein comprising only 43 amino acids, is widely assumed to parallel the structure–function relationships of its SARS-CoV ortholog based solely on sequence homology. In this study, we challenge this paradigm through direct physicochemical and structural characterization. Sequence analysis and electrostatic profiling reveal that the SARS-CoV-2 protein is a macromolecular polyanion with a net charge of −4 at neutral pH, featuring a diffuse negative surface that is highly responsive to pH changes. Complete 3D structures generated via ab initio modeling display a helical core flanked by two highly fluctuating, disordered termini. Residue Interaction Network (RIN) topology and Normal Mode Analysis (NMA) identified specific hinges governing these flexible extremities. Furthermore, the calculated dipole moment vector is tilted outward by 24°, misaligning with the central axis. Molecular dynamics simulations suggest that while the soluble structure is highly stable in water, it undergoes severe distortions and insufficient solvation within a membrane-mimetic environment. Thermodynamic association profiles and verified interactomic data from BioGRID reveal a strong propensity for ORF7b to participate in liquid–liquid phase transitions alongside human and viral partners. Taken together, these unique properties suggest that ORF7b operates as a dynamic peripheral membrane protein rather than a sedentary transmembrane component, providing a fresh framework for future therapeutic targeting. Overall, these in silico findings shift the current paradigm on ORF7b2 topology and provide a robust, physically grounded framework that identifies specific molecular priorities for future in vitro and in vivo validation.

## 1. Introduction

ORF7b-like folds belong to a protein family designated as “Nonstructural proteins 7b, SARS-like” (IPR021532) found across human coronaviruses [1,2]. While these sequences display no significant global homology to the human proteome [3,4], a notable seven-amino-acid sequence aligns with a portion of the human olfactory receptor, potentially providing a mechanistic link to virus-induced anosmia. Crucially, such interactions with extra-Golgi host components, combined with the evolutionary conservation of ORF7b folds, suggest a highly specialized and active role in viral biology rather than a sedentary structural function [5]. Despite its importance, neither a detailed experimental 3D model nor a comprehensive physicochemical characterization of the ORF7b fold has been established to date.

In SARS-CoV-2, the accessory protein ORF7b (herein ORF7b2) comprises 43 residues [1], one fewer than its ortholog in SARS-CoV (ORF7b1). Although they share 85.4% sequence identity and 97.2% similarity, they differ substantially in their charged amino acid composition [2,6,7]. Accessory proteins are widely recognized as non-essential for viral replication but critical for modulating host pathogenesis [8]. To date, much of the structural and functional literature on ORF7b2 relies heavily on historical assumptions of strict functional equivalence derived from pre-2018 studies of ORF7b1, including the foundational work of Pekosz et al. [1,9,10].

However, emerging evidence reveals that their interaction profiles diverge significantly, challenging this paradigm of functional conservation. High-throughput data filtering via BioGRID (COVID-19 Coronavirus Curation Project) indicates that while ORF7b1 interacts effectively with only 12 viral proteins, ORF7b2 targets a broader network encompassing 9 human and 12 viral proteins (see Table S1), with only 4 partners shared between the two orthologs. This expanded host interactome is supported by independent Affinity-Purification-Mass-Spectrometry (APMS) analyses, which identified over 300 high-confidence interactions between SARS-CoV-2 and human proteins, underscoring the multifaceted regulatory capacity of ORF7b2 [11]. For these physical interactions to occur in a crowded intracellular environment, the molecules must possess fine-tuned affinities and compatible spatiotemporal distributions.

This functional divergence is further mirrored by conflicting cellular localization data. Early literature localized ORF7b1 predominantly to the Golgi apparatus [9,12], whereas recent studies have identified ORF7b2 within the endoplasmic reticulum (ER) [13] or broadly dispersed throughout the cytoplasm [14]. This spatial fluidity suggests a multi-compartmental role affecting various metabolic pathways far from the Golgi matrix [8,11,14]. Solid-state NMR and structural interpretations remain biased by experimental conditions; prior studies characterizing the alpha-helical region (residues 8–29) used surfactants (e.g., DDM, SDS) or deuterated water (D2O) [15,16], which artificially stabilize helices and distort native conformations [17,18,19]. Crucially, these structural models often exclude the terminal tails, neglecting over 51% of the total protein sequence and leaving structure-function relationships speculative [16].

Our recent interactomic modeling confirmed that ORF7b2 is a multifunctional agent involved in over 5000 functional terms across diverse cellular environments, spanning cell-surface signaling, immune modulation, and tissue-specific metabolic pathways [8,20]. It demonstrates a remarkable capacity to engage in one-to-one interactions with structurally heterogeneous human partners (e.g., LPAR1, RPS4Y2), implying that the protein must dynamically adapt to microenvironments with markedly different chemical and physical properties [21,22]. Rather than acting as a sedentary transmembrane anchor [23,24,25,26,27], ORF7b2 exhibits the hallmarks of a mobile, adaptable peripheral protein.

Miniprotein structural dynamics are inherently linked to their local physicochemical fluctuations, which dictate conformational transitions [28,29,30,31,32]. Therefore, a holistic approach that accounts for the entire primary structure, especially the long, charged terminal regions, is necessary to explain how this polyanionic system operates across different cellular compartments. These features enable transitions between conformations, as structure is intrinsically linked to function. However, these transitions vary in time and space, and cellular function, the local environment, and molecular interactions influence their course [33,34,35]. Therefore, regardless of where ORF7b-like structures operate within the cell, they must possess the physical and chemical characteristics necessary to perform their functions [36,37]. ORF7b2 should also follow this rule.

This study aims to resolve the structural and functional mechanisms of ORF7b2 by presenting complete 3D models and executing a rigorous computational pipeline. We analyze its sequence topology, electrostatic surface properties, pH-dependent net charge variations, and conformational flexibilities via Residue Interaction Networks (RIN) and Normal Mode Analysis (NMA). We model molecular dynamics in explicit water and lipid bilayers, comparing its behavior to ORF7b1 to highlight how its unique physicochemical attributes are both sufficient and necessary to drive biological roles and potential participation in biomolecular condensate formation. ORF7b2 is a tiny protein, making it intrinsically difficult to characterize using conventional structural and functional approaches. In our work, we address this limitation by systematically analyzing its physicochemical properties and integrating these characteristics into a coherent computational framework. The proposed model appears robust and identifies specific molecular aspects that should be prioritized for experimental validation to clarify the functional role of ORF7b2 in the viral life cycle and host response.

## 2. Results

### 2.1. Sequence

The 3D structure of ORF7b2 has not yet been determined experimentally. As a result, we have a limited understanding of structure-function relationships because we do not know how its structure behaves in the biological environments where it operates. We aim to identify which structure-function relationships are attributable to ORF7b2 by comparing its properties with those of ORF7b1, when necessary.

Comparing the two proteins (Tables S2 and S3) helps clarify how similar they are and how their functional behavior might be similar. Figure S1 shows the distribution of hydrophilicity along both proteins. The central 21-residue segment (9–30) is hydrophobic and similar in both proteins; however, all tails are strongly hydrophilic and rich in charged residues. Table S2 also shows that ORF7b2 lacks positively charged residues and proline, whereas ORF7b1 contains one proline and a positively charged residue. In Table S3, we see ORF7b2 residues with high disorder propensity [38], such as T, A, D, H, Q, S, and E in the C-terminal, and E, S, D in the N-terminal, where D is also a known helix-disrupting residue [39]; however, disorder is common in globular and transmembrane proteins [40]. ORF7b1 has a proline at position 40 at its C-terminus; proline is another helix-disrupting residue. The sequences suggest that the C-terminal segments may fluctuate due to reduced or absent local helical organization.

### 2.2. Electrostatic Properties

#### 2.2.1. Conformational Analysis According to Pappu

**Figure 1:**
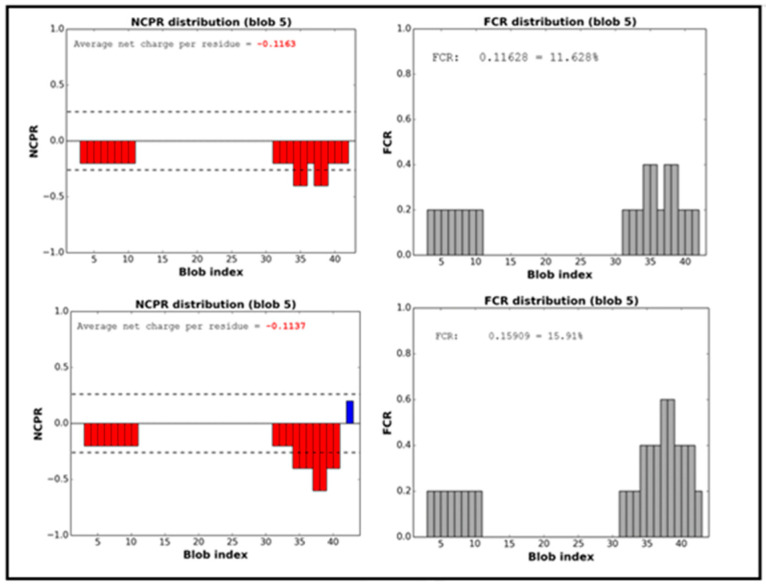
Distribution of ORF7b2 (top) and ORF7b1 (bottom) electrical charges. NCPR is the net charge distribution per residue (positive in blue and negative in red), and FCR, the fraction of charged residues. The proteins exhibit a widespread negative surface charge, with fractions of charged residues (FCR) in both terminal segments and a notable asymmetry in their charge distribution (sigma values) due to the negatively charged terminals. The high charge intensity on the tails facilitates the diffusion of negative charges throughout the entire structure. In fact, the overall charge distribution (NCPR) is negative for all residues on average.

**Figure 2:**
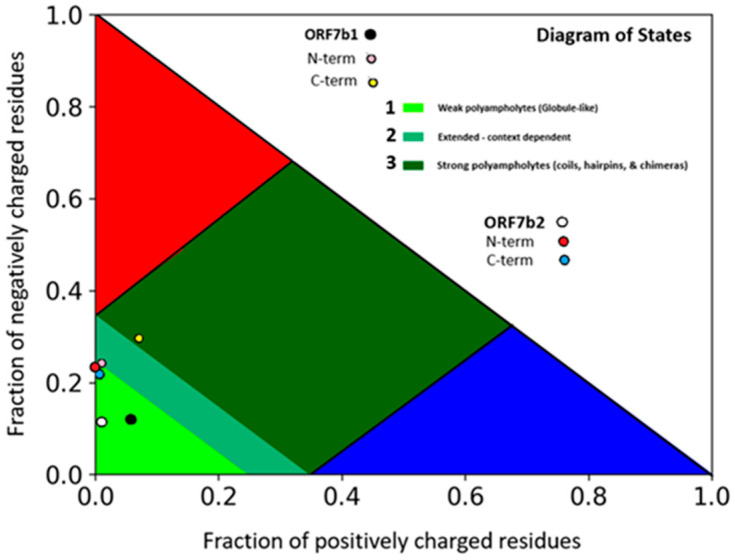
The state diagram shows ORF7b1 (black circle) and ORF7b2 (white circle). Both ORF7b1 and ORF7b2 are weak polyampholytes in region 1, exhibiting a propensity for a globular structural architecture with a low FCR and a negative NCPR (Table 1). Segmental analysis reveals different tail tendencies compared to full-length proteins. The tails of ORF7b1 adopt organizations that persist in regions 2 and 3. In particular, the C-terminus is in region 3 and features a coiled-coil hairpin structure, characterized by an FCR of 0.357 and an NCPR of −0.214. An FCR < 0.25 and an NCPR between −0.25 and −0.23 characterize the elongated globular shape of the ORF7b2 tails. The red region includes polyelectrolytes with a strong negative charge, while the blue region includes those with a strong positive charge.

The primary structures were analyzed using the Das-Pappu state diagram framework [41,42] to predict solution conformations based on electrostatic charge distributions (Table 1). Both orthologs feature negative net charge distributions per residue (NCPR). However, they differ in their fraction of charged residues (FCR), with ORF7b1 exhibiting a more asymmetrical distribution. We have also focused on the electrostatic properties [43]. The analysis of the charge distribution of the two proteins (Figure 1) reveals somewhat similar negative net charge per residue (NCPR) values, but differing charged residue fractions (FCR), with a more asymmetrical distribution for ORF7b1. In the diagram of states [42], both full-length proteins map strictly inside Region 1 (Figure 2). This classification identifies them as weak negative polyampholytes with a propensity for compact, globular/tadpole-like conformations in solution.

Segmental analysis (Figure 2) reveals a marked topological divergence between the flanking tails. The N- and C-termini of ORF7b2 remain inside Region 1, favoring an elongated globular architecture. Conversely, the tails of ORF7b1 shift into Region 2 and Region 3; its C-terminus, specifically, acts as a strong polyampholyte (FCR = 0.357), (NCPR = −0.214) with a predicted coiled-coil hairpin topology. The high surface charge density and the overall negative net charge (average NCPR of −0.1163 for ORF7b2) suggest a massive thermodynamic barrier for membrane insertion (Figure 1 and Table 1). Solvating these dense negative charges within the hydrophobic, low-dielectric core of a lipid bilayer is energetically unfavorable. These severe electrostatic constraints indicate that ORF7b2 may function outside a sedentary transmembrane state, a critical biophysical property overlooked in prior structural models (Figure S2).

Polyampholytes, according to reference [45], have a low net charge per residue but a high fraction of positively and negatively charged residues. Therefore, the behavior of ORF7b2 in solution should be that of a weak negative polyampholyte (FCR < 0.3), which should behave as an extended system with negative charges asymmetrically distributed in both terminal segments (Figure 1). The entire protein also carries a distributed negative charge, averaging −0.1163 net charge per residue. ORF7b1 functions as a weak polyampholyte, but its charge distribution is more asymmetrical than ORF7b2’s (Table 1), and it has a comparable mean NCPR. The small size and limited total surface area characterize these small proteins, favoring a measurable surface charge density distribution, a consequence of their strong overall negative charge.

Analyzing these results requires careful consideration of ORF7b2’s transmembrane location due to the negative charge on both leaflets of the phospholipid bilayer at physiological pH [46,47,48]. A net negative charge per residue extending throughout the structure, negatively charged terminal regions, and the high energy required to solvate negative charges (such as those of aspartic or glutamic acid) in the nonpolar interior of the membrane represent major obstacles.

#### 2.2.2. Dependence of Net Charge on pH

To understand the behavior of both proteins in solution, we calculated the dependence of the net charge on pH (see Section 4 for details).

**Figure 3:**
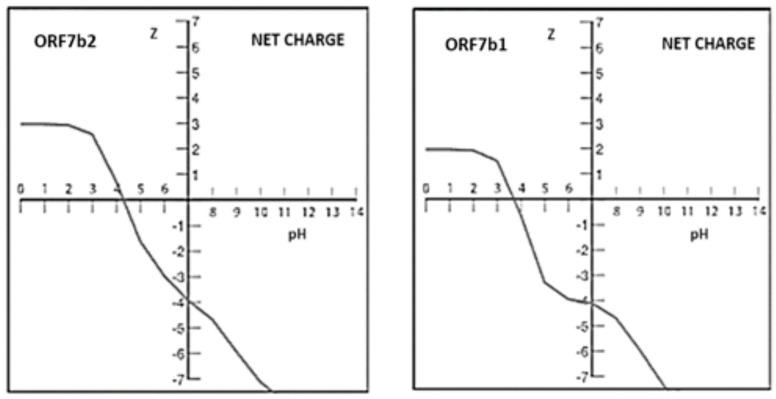
The dependence of the net charge (Z) on pH. The figures show that, at neutral pH, ORF7b1, and ORF7b2 have a negative net charge (Z = –4.2 and –3.8). Both curves became positively charged above pH 3 but remained negatively charged at high pH.

The calculated net charge (Z) of both proteins varies markedly with pH (Figure 3). At neutral pH, ORF7b1 and ORF7b2 possess net negative charges of −4.2 and −3.8, respectively. Both titration curves feature remarkably steep slopes within the physiological range, indicating high environmental sensitivity. ORF7b2 crosses neutrality at an isoelectric point (pI) of ~3.8, while ORF7b1 displays a distinctive curve shoulder centered at pH 6.0, crossing neutrality at pI ~4.2. This electrostatic responsiveness allows both proteins to modulate their surface charge profiles dynamically between approximately +3 and −7 across a pH range of 3.0 to 10.0.

Even within the physiological range, the steep slope of the curve alters the net charge and its distribution on the surface. These changes significantly influence the electrostatic interactions that the two proteins can engage in with other proteins or membranes, promoting diverse cellular activities. That ORF7b2 has many physical interactors (see Table S1) suggests it must have mechanisms to access and interact with these proteins in multiple cellular compartments. Its ability to modulate net charge broadens its interaction potential, explaining ORF7b2’s involvement in various metabolic activities across diverse cellular environments.

Segmental profiling identifies the C-terminal tails as the primary drivers of the differences in these curves (Figure S3). While the identical central hydrophobic cores maintain a net charge of zero between pH 3.5 and 6.5, the C-terminal region of ORF7b1 introduces a negative shoulder due to the cumulative titration of scattered carboxylic groups. This high electrostatic responsiveness, particularly in ORF7b2, underpins its capacity to engage in versatile interactions across distinct, non-membrane-bound cellular compartments, eliciting specific functional responses.

#### 2.2.3. Thermodynamic Stability and Surface Polarity Maps

To evaluate how environmental variations impact folding stability, atomistic solvent-accessible surface area (SASA) [49,50] was combined with Debye-Hückel electrostatic calculations [51,52,53,54]. Atom-by-atom profiling reveals that while both orthologs share a predominantly apolar central core, they diverge drastically in their terminal surface properties (Figure S4). ORF7b1 features relatively hydrophobic termini, whereas ORF7b2 displays a distinctly polar C-terminal tail and a highly charged N-terminus. These differences indicate that the two proteins exhibit significantly different surface charge distributions, reflecting distinct local physicochemical properties that could influence molecular interactions and functional behavior.

**Figure 4:**
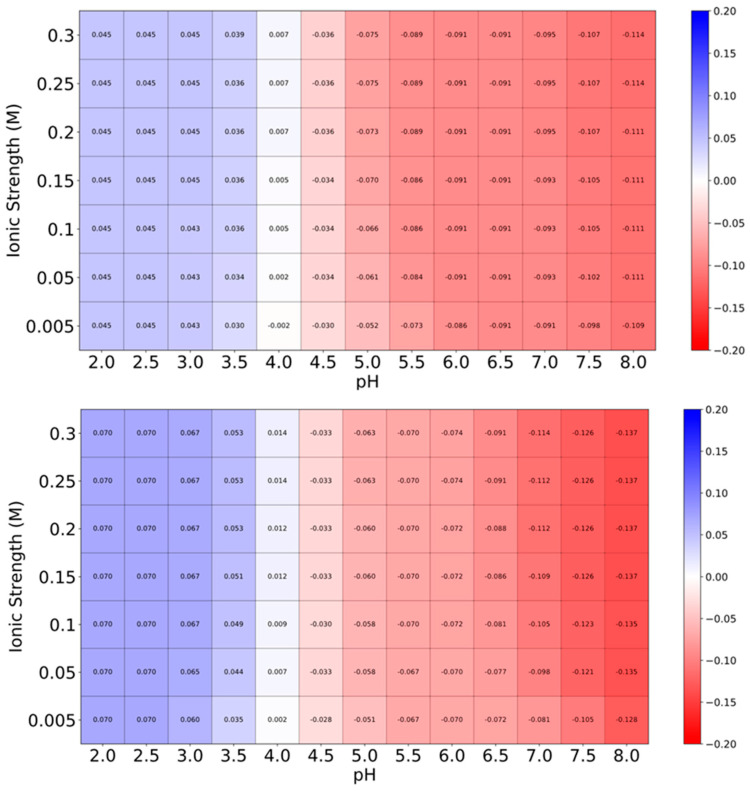
Charge Heatmap (e/residue) for ORF7b1 and ORF7b2. Net charge distribution per residue as ionic strength and pH vary for ORF7b1 (**top**) and ORF7b2 (**bottom**). The color scales on the right show the correlation with the charge values.

**Figure 5:**
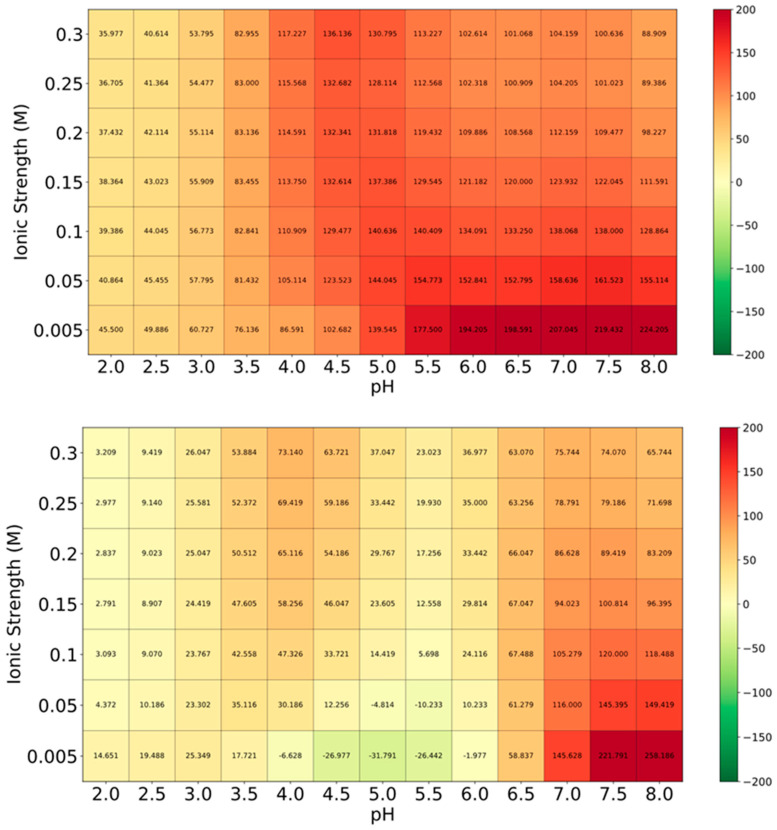
Energy heatmap (Joules/residue for ORF7b1 and ORF7b2. The energy distribution per residue as ionic strength and pH vary is shown at the (**top**) for ORF7b1 and at the (**bottom**) for ORF7b2. The color scales on the right indicate the correlation with the energy values.

This divergence is also reflected in the per-residue charge and energy distribution heatmaps under varying pH and ionic strength conditions (Figure 4 and Figure 5). For ORF7b1, the calculated energy contributions remain uniformly positive across all conditions, peaking at alkaline pH and low ionic strength. This profile indicates thermodynamic stability and high solubility strictly in apolar media, corroborating its role as an intrinsic membrane protein.

Conversely, the energy landscape of ORF7b2 is substantially different. Between pH 4.0 and 6.5, absolute energy values drop toward zero and become negative at low ionic strength; a stability maximum is observed at pH 5.0. This clear window of stability in polar environments suggests that ORF7b2, unlike its ortholog, can exist in a stable, soluble state within aqueous systems. This thermodynamic behavior aligns with the maximum slope of its titration curve, confirming that minimal pH fluctuations between 4.0 and 6.0 govern the structural partitioning and interaction potential of ORF7b2.

### 2.3. Structural Properties

#### 2.3.1. Complete 3D Modeling and Conformational Ensembles

**Figure 6:**
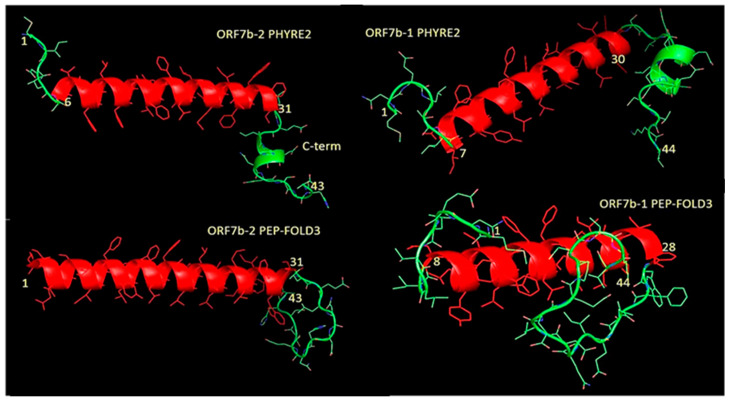
The figure shows the best models from two prediction platforms, PHYRE2 and PEP-FOLD3. Both use templates to predict the central helical segments [red] and ab initio methods for the terminal segments [green]. We assume the folding process occurs at neutral pH (see Supplementary Materials for details). PyMOL (Open Source, version 2.5.0) provides structure visualization (https://pymol.org/2/ accessed on 28 June 2026).

To overcome the limitations of truncated literature models, full-length 3D structures for ORF7b1 and ORF7b2 were generated via PHYRE2 [55] and PEP-FOLD3 [56,57,58] platforms, achieving an overall model reliability of 88% (Figure 6). They modeled the central helical residues using specific templates (Tables S4 and S5), while modeling the outer C- and N-terminal segments (highlighted in green) using ab initio techniques. Both platforms converged on a similar structural architecture: a compact central helical core flanked by highly fluctuating, non-helical terminal segments. The C-terminal tail is notably longer (12–14 residues) and more conformationally varied than the N-terminus.

The charge distribution analysis presented in Figure 1 shows an asymmetric distribution of negative charges on the proteins, and the three-dimensional models reflect this asymmetry. One of the most widely recognized ORF7b2 models is from ModBase (University of California, San Francisco–UCSF) (Figure S2). Like many others, this model shows only the 3D structure of the region between Leu4 and His37, which is predicted to be a helix, and omits all terminal residues.

**Figure 7:**
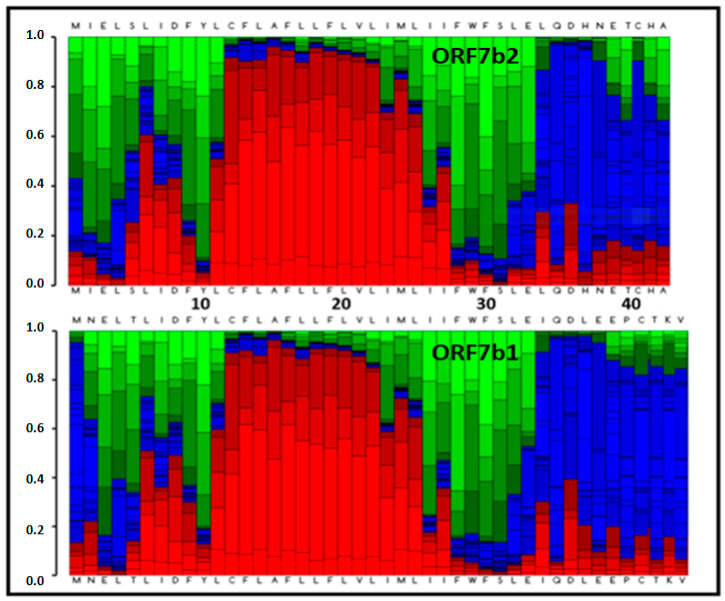
Conformational probabilities. The graph shows the conformational probabilities (ranging from 0 to 1) for each residue in the two proteins, as predicted by PEP-FOLD3. The graph displays the probabilities (on the vertical axis) for each sequence position (on the horizontal axis). PEP-FOLD3 is based on the concept of a structural alphabet [59], in which an ensemble of elementary prototype conformations describes the full diversity of protein structures. Each residue is represented as the average of 4 residues. The profile uses the following color codes: red for helical, green for extended, and blue for coil. The graphs illustrate how charges on terminal residues influence conformation, with extended structures prevalent at the C-terminus and coil formation at the N-terminus. For a more detailed comparison with the predicted 3D structures, see Figure S5.

However, we can gain a more detailed understanding by analyzing the conformational probabilities of each residue in the two proteins. This analysis, performed by PEP-FOLD3, is based on the concept of a structural alphabet [59] and determines the average weight of each elemental conformation that each residue adopts to define the protein’s structure. Figure 7 shows the weighted distribution of all conformations for residues [57,59] in both proteins. This statistical analysis shows that the compact helical core spans only 11–12 residues. The flanking regions exhibit a structurally heterogeneous ensemble. At both N-termini, there is a marked preference for extended and coiled states rather than a single rigid fold. The long C-terminal segments undergo a specific localized transition, populating predominantly extended states between residues 26 and 33, before switching to a dominant coiled conformation from residue 33 to the C-terminus. This high structural plasticity facilitates continuous terminal fluctuations, which likely modulate dynamic local interactions. From a conformational perspective, the two proteins have a compact helical core of 11–12 residues, which is structurally insufficient to span a standard 20-residue lipid bilayer [60,61].

An interesting pattern emerges in the overall conformational behavior of the residues defining the terminal segments of the two helices. A closer examination of the weighted conformational distribution at both N-termini reveals that the extended and coiled states (shown in green and blue in the figure) account for a substantial proportion of the sampled structures, with the extended conformation contributing more prominently. This indicates that the N-terminal regions do not adopt a single rigid arrangement, but populate a structurally heterogeneous ensemble with a marked preference for more extended states.

The two C-terminal regions show broadly comparable behavior, although they differ in the relative abundance of the coiled and extended conformations. In particular, residues 26 to 33 are characterized predominantly by the extended conformation (green), whereas from residue 33 onward to the end of the segment, the coiled conformation (blue) becomes dominant. This shift suggests a conformational transition along the C-terminal tail, with distinct local preferences emerging across different residue ranges.

Considering that the weighted conformational profiles of the individual residues in both terminal tails remain quite variable, this supports the view that these regions retain a considerable degree of local flexibility. Such variability implies that the corresponding segments may be prone to adopting multiple structural organizations, rather than remaining locked in a single, well-defined arrangement. In this context, the data are consistent with a dynamic interconversion between extended and coiled states, likely reflecting an intrinsically mobile character of the terminal regions.

The N-terminal segment likewise appears flexible, although it displays a stronger tendency toward an extended organization. Overall, these observations suggest that the terminal portions of the helices are best described as non-helical, conformationally dynamic regions where the residues are likely to undergo continuous fluctuations. This structural plasticity may be functionally relevant, as it could facilitate transient rearrangements, modulate local interactions, or contribute to the adaptability of the helix termini within the broader molecular context.

#### 2.3.2. Ramachandran Analysis

Ramachandran diagrams of both models, ORF7b2 and ORF7b1, illustrate in more detail some points already discussed [62]. The non-helical nature of the terminal segments was confirmed through backbone dihedral angle mapping (Figure S6a,b). Both orthologs display numerous terminal residues with Phi and Psi angle combinations that cluster outside the core alpha-helical region, mapping instead to extended, beta-sheet, or sterically outlier zones (e.g., Glu3 and Leu20 in ORF7b2; Leu6 and Trp29 in ORF7b1).

Crucially, the density of residues occupying canonical helical angles (Phi is approximately 60° and Psi approx. −50°) differs significantly between the two proteins. ORF7b1 features a well-populated, contiguous helical block that includes residues Phe9, Cys12, Phe13, Phe16, Leu17, Phe19, Val21, Ile23, Leu25, Leu26, and Phe28. In contrast, ORF7b2 displays fewer helical residues (Phe13, Leu17, Leu18, Val21, Leu22, and Leu25), showing a shorter core helix that is subject to structural interruptions.

These stereochemical data, combined with the charge profiles, show that the terminal segments are neither helical nor embedded in the lipid bilayer. Instead, they remain highly mobile and solvent-exposed, suggesting that ORF7b2 possesses unique structural traits that favor flexible extra-membrane localization or unconventional membrane-interaction modes.

The charge distribution analysis shows that the tails of both proteins are neither helical nor embedded in the membrane. The modeling systems also support a non-helical structure that is likely flexible and mobile. Although this aligns with the overall view, the distribution of helical residues in the Ramachandran plots varies. Overall, ORF7b2 exhibits many structural features beyond those of a transmembrane protein, regardless of ORF7b1’s traits. While we cannot rule out its involvement in membranes, ORF7b2 exhibits chemical, physical, and structural properties that suggest it may be located elsewhere in the cell or interact with membranes differently.

#### 2.3.3. The Representation of Non-Covalent Interactions by Graph Theory: Residue Interaction Network (RIN) Analysis

**Figure 8:**
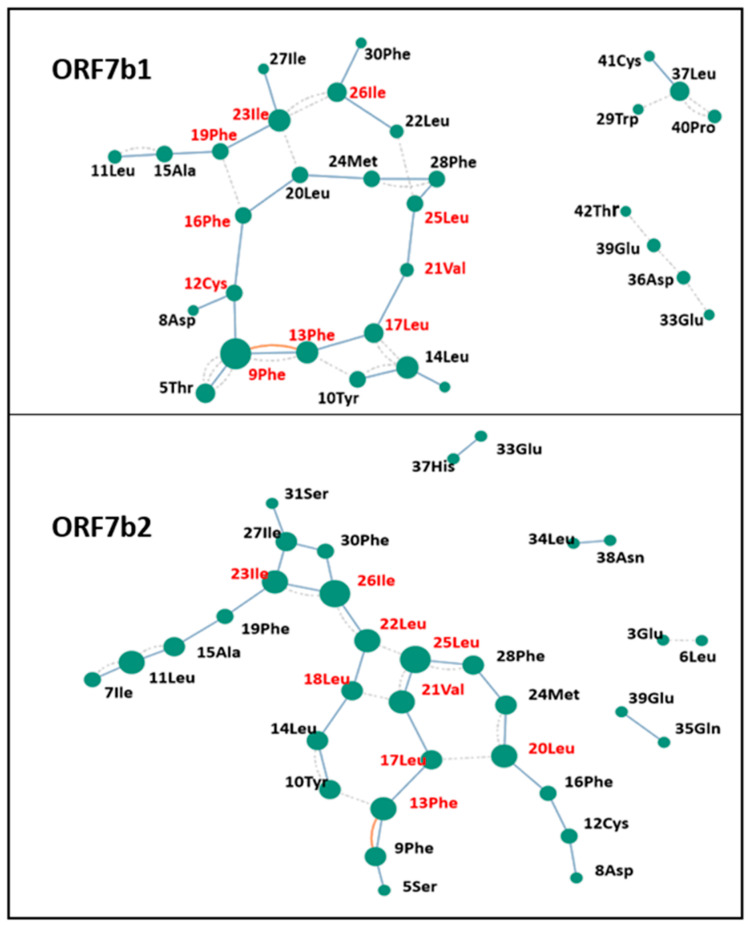
The figure illustrates the molecular contact networks of ORF7b1 and ORF7b2, generated by RING4. In the graphs, nodes represent residues, and edges depict weak molecular interactions. The analysis excludes covalent bonds between residues to better visualize weak interactions. We assessed contacts using hydrogen bonds or van der Waals interactions between residues (Table S6). The residues that are most topologically important and play a key role in structural coordination are highlighted in red. Dashed lines show van der Waals interactions, while solid lines represent hydrogen bonds. The red curved line between 13Phe and 9Phe illustrates a π-π stacking interaction. We identified these residues using Cytoscape to calculate betweenness centrality (Table S7). Residues that are unconnected to the interaction network are omitted. We used Cytoscape to visualize these networks and the unconnected residues (Figure S7), as determined by RING 4.

To investigate intra-chain stabilization and information propagation at the single-residue level, probabilistic Residue Interaction Networks (RINs) were generated via RING 4.0 (Figure 8). Non-covalent interactions (hydrogen bonds, van der Waals forces, and π-π stacking) were mapped (Table S6) using specific distance cutoffs, and topological importance was quantified via Cytoscape (version 3.10.3) by calculating betweenness centrality values (Table S7). More central nodes exert a greater influence on the transmission of information between other nodes in the network. Peripheral residues, with fewer connections, represent mobile and flexible regions.

A protein is composed of residues (or groups of residues) that exhibit specific contact patterns. Information is transmitted among structural clusters (or subclusters) interested in it, thereby minimizing energy and stabilizing the structure. Therefore, treating the protein as a single network of uniform interactions can lead to inaccurate conclusions and predictions about the system’s fundamental dynamics. This is consistent with the energetics associated with the geometry and topology of hydrogen bonds in helices, which, although similar in appearance, exhibit different energetic stability coefficients for each bond.

The most accurate approach is to identify structural clusters by tracking their interaction patterns through a multilayer approach [63]. This method also allows us to assess the importance of individual residues or residue groups within the protein using topological metrics such as betweenness centrality. As we will see, ORF7b1 and ORF7b2, two seemingly similar proteins, differ significantly in their structural organization.

RIN representation converts 3D protein structures into graphs (interactome) and applies graph learning to infer interactions [64,65,66,67]. The RIN analysis identifies the physicochemical nature of non-covalent interactions at an atomic level within protein structures [68]. Each residue is represented as a node in the graph, and interactions between nodes are encoded as edge features. Residues are dynamically connected to each other to enhance message flow between subgraphs [69]. Information propagation between nodes depends on the topology of native contacts, allowing neighboring nodes to exert different levels of influence on a central node, from small atomic fluctuations to collective movements of entire domains, subunits, and molecules [70]. In a single helical structure, intramolecular interactions, which depend on the features of the 3D structure, dominate the motions and are structure-encoded [71]. The native contact topology plays a key role in defining local collective movements and lends itself well to analytical approaches that describe the collective modes of specific architectures [72]. RING4.0 processes multistate structures, such as protein “conformational states,” from PDB files, using molecular dynamics and structural ensembles to identify noncovalent interactions at the atomic level [73,74]. Thus, the dynamics of each interaction are considered in the context of the entire structure.

Topological mapping [75,76] revealed a stark divergence in network architecture and rigidity between the two orthologs:

ORF7b1 Network Topology: ORF7b1 exhibits a highly interconnected, rigid core that is distributed continuously across the central helical domain from residues 9 to 26 (Figure 8, Tables S6 and S7). This core is structured into three contiguous, overlapping sub-graphs stabilized by high-centrality “Hub” residues (e.g., Cys12, Phe16, Phe9, and Ile23). This extended network of non-covalent constraints rigidifies the helical core [77], structurally supporting its function as a classic transmembrane anchor.

ORF7b2 Network Topology: Conversely, the rigid network of ORF7b2 is significantly shorter and restricted strictly to residues 17 to 26 (Figure 8, Tables S6 and S7). The central domain features two distinct network breakpoints (at positions 14–16 and from residue 27 onward) where stabilizing non-covalent interactions are entirely absent. Within this interrupted framework, Phe13 appears isolated from the core network, engaging only in a local π-π stacking interaction with Phe9 to modulate their relative orientation.

In both models, approximately half of the total residues fail to engage in weak chemical bonding with the central core, forming small, independent, or completely disconnected sub-graphs (Figure 8). These unconstrained peripheral segments match the structural boundaries of the flexible tails. However, while the mobile extremities of ORF7b1 flank a highly compact, sedentary core, the shorter, rigid network of ORF7b2 permits a substantially wider range of local and collective motions. This elevated structural flexibility, driven by an interrupted contact topology, underpins ORF7b2’s capacity to dynamically transition between cellular microenvironments.

Interestingly, all the alpha-helical residues identified in the Ramachandran plots are crucial in both proteins, underscoring the importance of the central helical segment for their stability. The graph in Figure S7 illustrates the numerous unconnected residues and shows the organization of the compact structure containing the critical residues, as shown by Cytoscape. The absence of weak molecular interactions in about half the residues of both molecules suggests that the less stable, more flexible regions are extended.

**Figure 9:**
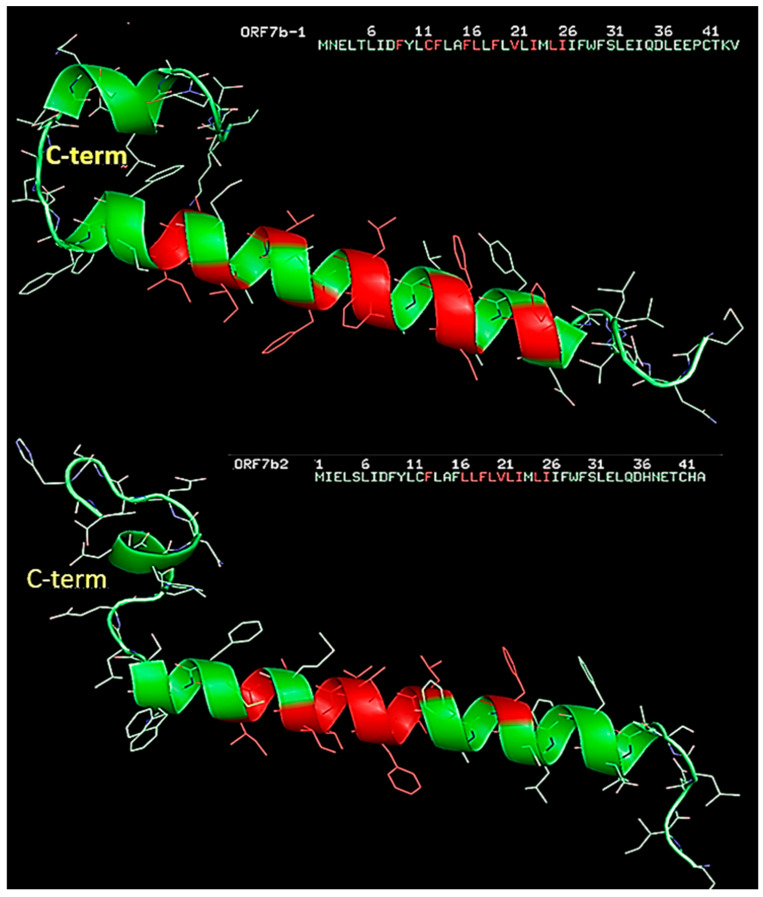
Comparison of ORF7b1 and 2 structures with centralized residues highlighted in red. The legends in the figures report the sequences, with the central residues in red corresponding to those in the structure.

To visually impact the architectures, we mapped high-centrality residues onto the three-dimensional structures of the two proteins (Figure 9). The residue distribution reveals two distinct structural organizations, which allow us to interpret our results. ORF7b1 exhibits a well-organized distribution within the central helical segment, forming a compact network from residues 9 to 26. Hydrogen bonds and van der Waals forces stabilize and stiffen the helical segment, supporting its function as a transmembrane helix [77]. The two tails are devoid of high-centrality residues, and many weak molecular interactions are absent, making them less constrained and more mobile. ORF7b2 shows a significantly different distribution. Its central segment, which contains the central residues, is visibly shorter and stabilizes and rigidifies the structure from residues 17 to 26. In the central helix, two breakpoints are present, at residues 14–16 and from residue 27 onwards, where stabilizing molecular interactions are lacking. Phenylalanine 13, which appears isolated, forms the π-π stacking interaction with phenylalanine 9, likely helping to stabilize their relative positions. Small, disconnected clusters, organized into independent subgraphs, are located in the C-terminal segment and exhibit local flexibility. Overall, ORF7b2 has a relatively small, rigid central segment, allowing greater structural movement; it is more flexible than ORF7b1, especially given the high mobility at both ends.

### 2.4. Dynamic Properties of ORF7b2

#### 2.4.1. Low-Frequency Vibrational Modes and Hinge Analysis

Most of a protein’s functional activities involve movements across a broad range of time scales, from very rapid motions (sub-picoseconds to microseconds) like conformational changes, segmental flexibility, and quick folding or unfolding, to low-frequency movements characterized by collective atomic fluctuations along structural hinges [78]. The collective fluctuations of weak bonds govern the dominant low-frequency mode, involving hydrogen bonds and the internal displacements of larger atoms. These low-frequency modes are part of the protein’s overall vibrational modes. Therefore, proteins can explore many conformations or undergo equilibrium fluctuations near their native structure. Normal mode analysis [NMA] is a useful method for characterizing the various dynamic aspects of proteins [79]. It examines proteins’ dynamic and structural properties by modeling vibrational modes, which often correspond to the slowest and most significant motions associated with the protein’s function [80]. Two web servers, elNémo (Network Elastic Model) [81,82,83] and HINGE-Prot [84], facilitated automated computational analysis of low-frequency normal modes [85]. Table 2 lists hinge residues with the highest scores, derived from conformational models that describe residue fluctuations from the average structure along the main directions of motion. The Gaussian network model [GNM] and anisotropic network model [ANM] [83] are used to compute HINGE-Prot models.

HINGE-Prot analysis identified residues Leu20, Phe9, and Leu32 as critical structural hinges governing global collective motions. The high-scoring Leu20 residue maps to the boundary of the core rigid cluster, coordinating local bending deformations through non-covalent interactions with Leu17 and Met24. Conversely, Leu32 acts as a distinct torsional hinge, linking the core to a rigid C-terminal subgraph (residues 23–31).

**Figure 10:**
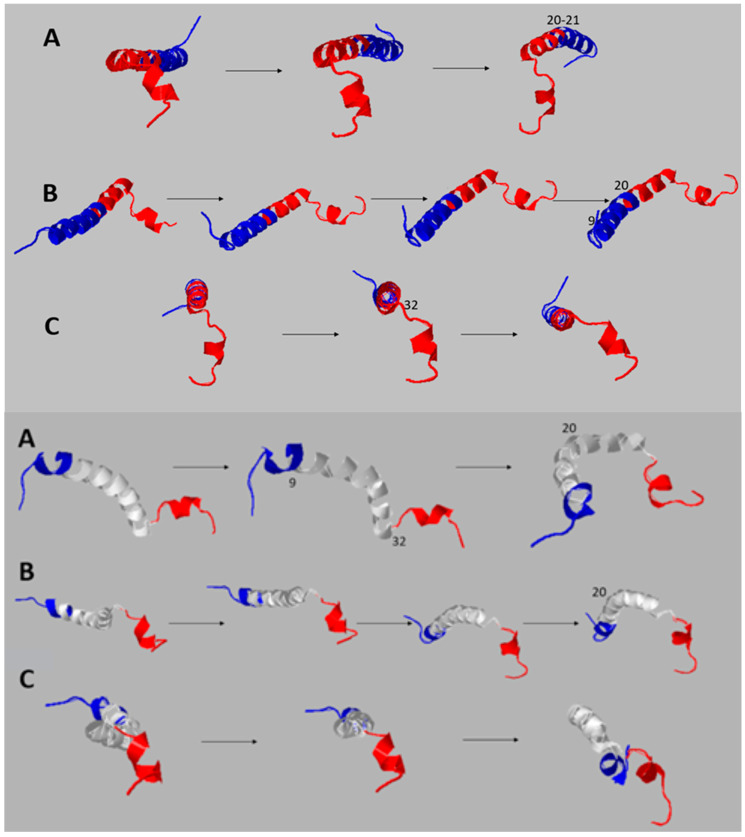
Dynamics around the hinge residues of ORF7b2 (see Table 2). The model displays the hinge position with the residue number. The figures present snapshots of motion from three different views (A, B, and C), and the arrows show the time sequence. (**Top**): Twist movements around residues 9 and 32. (**Bottom**): The backbone exhibits precise bending movements around residues 20–21. The different colors indicate different conformational segments of the protein.

**Figure 11:**
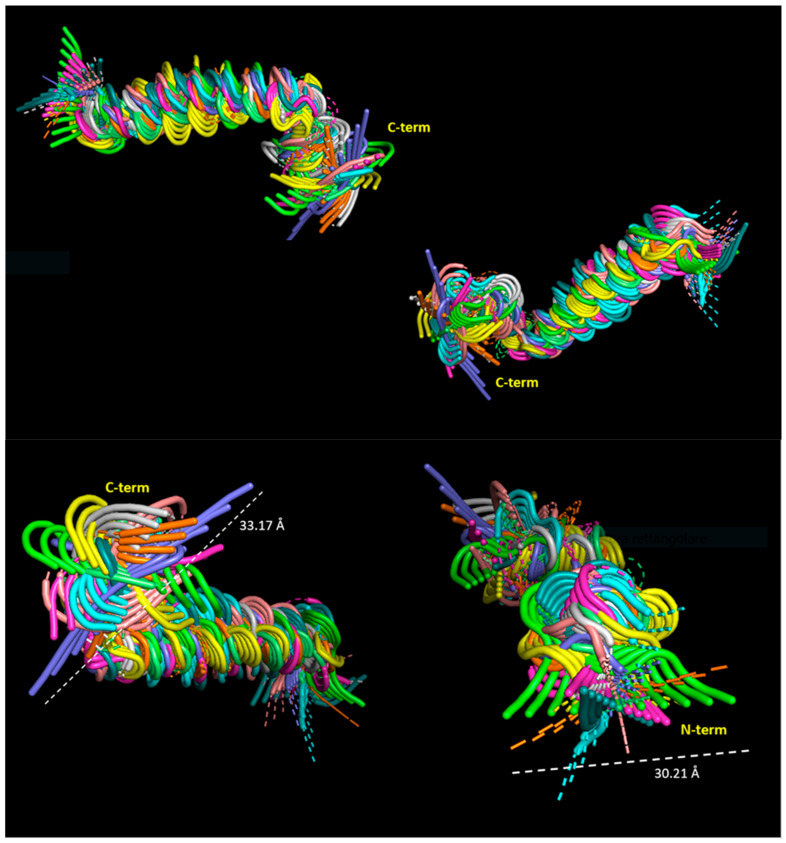
Local dynamics of ORF7b2—The superimposition of the normal modes reveals the set of local low-frequency molecular movements in ORF7b2. In the upper figure, there is a side view, while the lower figure shows a view along the molecule’s major axis. The central axis of the molecule vibrates (see also Figure S8) but remains quite organized, with minimal warping but apparent bending. In the bottom figure, both terminal segments exhibit large fluctuations and residue displacements of a few tens of angstroms. The different colors indicate the different conformations of the protein.

Vibrational mode animations track extensive twisting movements around residues Phe9 and Leu32, whereas Leu20 mediates precise backbone bending (Figure 10). Superimposition of the primary low-frequency modes reveals significant segmental displacement at both termini, with amplitudes on the order of several tens of Angstroms (Figure 11). In contrast, the central axis maintains a more structured alpha-helical fold, exhibiting localized bending and winding fluctuations restricted to 8–10 Å. These extensive degrees of freedom increase protein entropy and lower free energy, confirming that the dynamic flexibility of ORF7b2 readily accommodates stable non-membrane or peripheral membrane states without structural unfolding.

Likewise, residue 20Leu appears as a central component at the edge of a rigid cluster (Figure 10). This makes the evaluation of HingeProt quite reliable and logical. The covalent link between residue 32Leu and 31Ser, which connects to a rigid subgraph (23Ile-25Ile-30Phe-27Ile-31Ser), characterizes residues 31 and 32 as hinge points. The conformational fluctuations that cause residue twisting generate movements of entire regions.

Figure 10 also shows some motion sequences around the hinge residues of ORF7b2, generated by HingeProt calculations. The snapshots highlight the most notable twisting movements around residue 32. Residue 20 is involved only in bending movements. Although residue 9 is at the N-terminal segment, it is naturally mobile. Figures S8 and S9 report numerical values for several modes, indicating that the protein is extremely mobile at both ends, with a central segment characterized by marked fluctuations whose minima are in good agreement with those found by RIN. Figure S10 summarizes the main normal modes calculated for ORF7b2 using elNémo.

All of this supports the idea that we can explain the molecule’s overall flexibility in terms of its collective motions. The observed deformations fall into distinct modes: bending and twisting about the central axis, and torsional deformations at the ends of each α-helical segment (Figures S8 and S9). The observed structural irregularities show how involved they are in the overall motion of the molecule. These additional degrees of freedom increase the protein’s entropy, thereby reducing the system’s free energy and improving its stability. However, the dynamical modes of NMA about how α-helices behave as deformable bodies are similar between transmembrane α-helices, extramembrane α-helices, and α-helices in soluble proteins [86], since the deformations of α-helices are independent of cellular position [87]. Therefore, because ORF7b2 exhibits a wider range of segmental and terminal movements, this justifies its presence in environments other than membranes, provided these movements do not preclude interaction with nonpolar environments.

#### 2.4.2. Helix Macrodipole and Compactness

**Figure 12:**
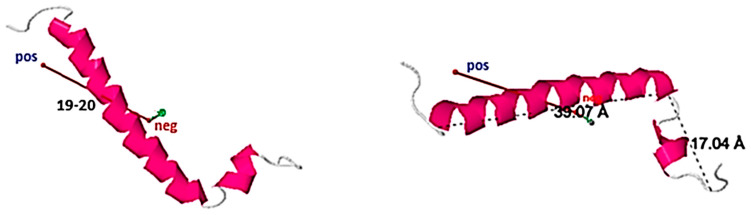
**Helix Dipole.** The ribbon diagram of ORF7b2 presents two views that highlight significant distortions in the dipole (red) and mass-moment (greenish) vectors. The center of mass is at residues Leu19-Val20, and the dipole vector points outward with a 24° tilt. Thus, it does not align with the protein’s central axis. The red dipole line’s origin coincides with the dipole moment’s overall negative charge, while its other end aligns with the net positive charge. Since the dipole is equivalent to a +0.5 charge at the N-terminus and a −0.5 charge at the C-terminus, missing positive residues near the C-cap end of the helix dipole destabilize the structure because of unfavorable interactions with harmful residues. This likely makes membrane insertion unstable. The distance in the figure estimates a central helix of 39.07 Å and a C-terminal mobile element of 17.04 Å. Both segments generate rotational solids converging into the molecule’s overall prolate ellipsoid.

Another parameter that provides insight into helix behavior is the helix macrodipole, also known as the helix dipole. It is a large-scale dipole moment present in all helices. This macro-dipole influences the helix structure, including helix packing, interactions with lipid bilayers, and charge distribution at binding sites [88,89]. We used the best three-dimensional structure from PHYRE2 for calculations on the server at http://bip.weizmann.ac.il/dipol (accessed on 10 March 2025) [90]. The server computed the dipole moment and displayed the dipole vector superimposed on a protein ribbon backbone (Figure 12 and Table S8).

The electrostatic alignment of individual peptide bonds yields a total Debye dipole of 488 D for ORF7b2, which falls significantly below the statistical average of 542.66 D reported for canonical helical proteins [90]. This deficit indicates a major structural misalignment driven by the asymmetric terminal charges and core distortions. The calculated dipole vector is tilted outward by 24° relative to the central helix axis, with the center of mass mapping to residues Leu19-Val20. Because the dipole lacks positive capping residues near the C-terminus, unfavorable electrostatic interactions destabilize the structure, rendering standard parallel membrane insertion unfavorable.

The calculated equilibrium radius of gyration Rg is 10.91 Å, which is substantially lower than the 19–20 Å expected for an ideal, fully elongated 43-residue alpha-helix [91]. This compact Rg value establishes that ORF7b2 adopts a condensed, prolate ellipsoidal geometry driven by intense terminal fluctuations. Crucially, the central core helix measures 39.07 Å, which significantly exceeds the hydrophobic width of standard lipid bilayers (~32 Å). Combined with the 24° dipole tilt, this hydrophobic mismatch forces a static membrane-inserted monomer to adopt an unstable 40° tilt angle relative to the bilayer normal, suggesting that membrane insertion induces severe structural distortions.

We present an attempt to visualize the insertion of a single ORF7b2 molecule into a membrane in Figure S11. Although the insertion pattern of ORF7b2 between two membrane layers appears static, this simulation shows a tilt angle of 40° relative to the axis normal to the membrane surface. To gather more details on the protein’s membrane insertion, we performed molecular dynamics simulations in water with a single molecule and in the membrane as a dimer.

#### 2.4.3. Molecular Dynamics of ORF7b2 in Explicit Water

**Figure 13:**
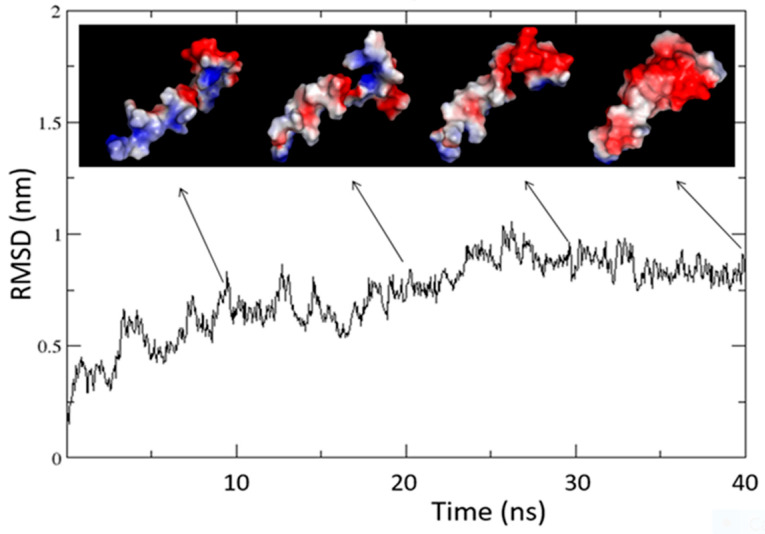
Molecular dynamics of ORF7b2. The figure illustrates the trend of the ORF7b2 molecular dynamics simulation in water. Around 25 ns, the protein reaches equilibrium. The simulation shows that the protein remains stable in an aqueous environment. The conformational adaptation toward the structural organization at equilibrium shows that the gradual conformational changes involved in settling produce electrostatic surfaces (negative in red, positive in blue, and hydrophobic in white) that vary in charge and extent. We calculated the electrostatic surfaces with DelPhi (see Section 4). The small size of the molecule shows how even minor conformational changes can easily influence its electrostatic surface.

To evaluate structural stability in a high-dielectric medium, atomistic molecular dynamics simulations were conducted in explicit water at neutral pH and 300 K. Minimized ORF7b2 reaches thermodynamic equilibrium at 25 ns and maintains structural integrity without unfolding (Figure 13). The calculated root-mean-square deviation (RMSD) stabilizes at ~1 nm (10 Å), consistent with the collective low-frequency fluctuations predicted by NMA.

During the simulation, subtle conformational transitions actively reshape the solvent-accessible electrostatic potential across the protein surface. At 40 ns, the equilibrium structure features a highly asymmetric charge topography: one face exhibits a dense, diffuse negative surface, while the opposite face displays an uncharged, hydrophobic core flanked by charged terminal tails (Figure S13).

This dynamic distribution directly disproves prior models in the literature that proposed a rigid “lysine zipper” (Lys4, Lys11, Lys18, Lys25) to support transmembrane anchoring. Our mobile simulation shows that these residues are fully dispersed across distinct electrostatic environments: Lys4 maps to the highly fluctuating N-terminus, while Lys11 and Lys18 are embedded within the large negative surface on the opposite side of the molecule. The intrinsic mobility of ORF7b2 thus dictates its surface charge distribution, enabling the protein to continuously modulate its electrostatic profile to capture diverse cytosolic or peripheral molecular partners.

**Figure 14:**
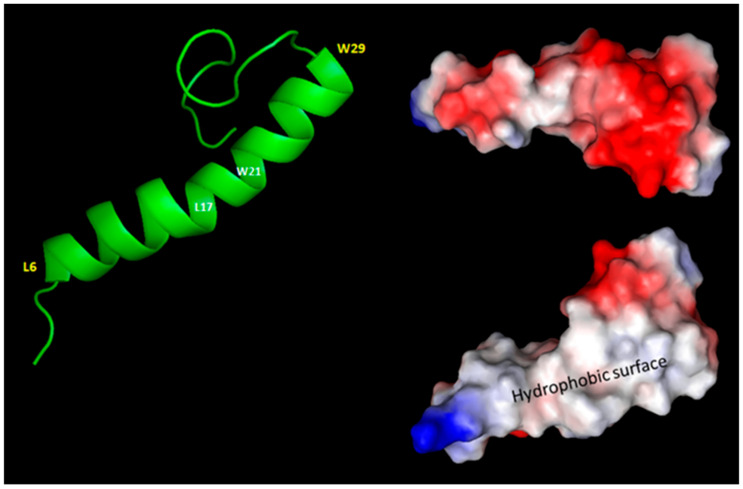
Main structural features of the ORF7b2 model obtained from molecular dynamics in water at neutral pH. The helix, which extends from L6 to W29, shows bending centered on residues L17 and W21. The surface representation shows that the two opposite sides of the protein have different electrostatic properties. A diffuse negative charge covers one side (in red), while the other side shows both charged ends (the positive charge in blue is from the NH3+ terminal), with the central surface predominantly hydrophobic (in white). PyMol displayed the electrostatic surfaces calculated by DelPhi [92].

In Figure S12, we show the time evolution of several molecular parameters. The figure depicts the RMSD (root-mean-square deviation) of atomic positions, with an equilibrium value of approximately 1 nm (10 Å), consistent with the results from the analysis of low-frequency molecular vibrational modes. This dynamical information supports our previous computational results for other molecular parameters. Physicochemical properties influence the solution behavior of the small molecule ORF7b2. During the simulation, conformational shifts result in structural changes in the protein. Parts of the protein rearrange with respect to each other, as evidenced by trends in helicity, hydrogen bonding, area per residue, and radius of gyration. Since this is a relatively flexible small protein, it is interesting to examine its surface electrostatic distribution [91]. Figure 13 also shows changes in the surface electrostatic distribution at 10 ns intervals during the simulation, with the surface electrostatic potentials calculated with the DelPhi program [92]. This software accounts for ionic strength effects when evaluating the Poisson–Boltzmann equation, as detailed in the Calculation Methods. During the simulation, the surface charge distribution of the protein changes, even with small conformational shifts, evidenced by changes in helicity and shape (Rg) (Figure S12). For example, the 40 ns equilibrium model in water (Figure 14) shows a positive charge distributed entirely on one side. This suggests that the stability of the protein in aqueous solution and its response to conformational changes depend on changes in the surface charge distribution, which likely influence the protein’s interactions with the solvent. This electrostatic behavior may also help guide its search for different molecular partners through basic interactions. Figure S13 provides more detailed views of the 40 ns conformation.

In the cartoon model (green), evidence indicates that, from L17 to W21, this segment acts as a pivot for the slight bending of the surrounding parts. However, in the top-right model, the distribution of electrostatic potentials on the protein surface shows that one side is negatively charged (red). In contrast, with a 180° rotation, the other side displays charges on both tails and presents an uncharged (non-polar) surface. Although this is a static view, it helps clarify the structural arrangement of the lysines, as some researchers have suggested that a lysine hinge supports the transmembrane localization of ORF7b1 [1,9,10] and ORF7b2 [16,26].

Regarding ORF7b2, the stripe (Lys 4, 11, 18, 25) proposed by Forgeon et al. [16,26] did not consider the structural motions of the protein nor its physicochemical characteristics. Instead, they relied on a static, correlation-free model. Their stripes do not align with these lines (Figure S13), but these residues are dispersed across charged surfaces. For example, lysine 4 is located at the N-terminal end, within a mobile charged region in transition between helix and ball, while lysines 11 and 18 are located on the opposite side of the molecule, embedded in a large surface with a diffuse negative charge. The high surface charge density of the small ORF7b2 makes these results predictable. Therefore, the molecule’s intrinsic mobility influences its electrostatic properties, which are related to the structural behavior, shape, position, and orientation of its residues.

#### 2.4.4. Dimer Molecular Dynamics Within the Lipid Bilayer

**Figure 15:**
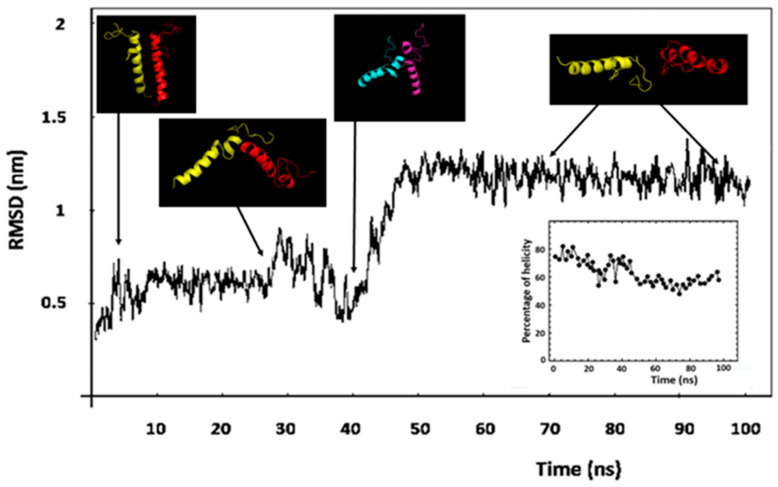
The trend of the molecular dynamics of the dimer in the membrane. For greater clarity, we show the structures at various times without the reference membrane (Figure S16). We used a model of the orf7b2 dimer in a parallel (cis) orientation. The graph shows the evolution of total helicity over the 100 ns simulation. The two graphs reveal, within the same time interval (35–55 ns), a nearly superimposable transition, suggesting a sudden change in structural organization, accompanied by a concomitant loss of helicity and an increase in the average interatomic distance across the system. We extended the dynamics up to 200 ns without observing any changes compared to this experiment.

Some authors suggest that ORF7b2 forms multimeric organizations within membranes, but the protein’s ability to do so remains unclear. To test prior hypotheses, atomistic molecular dynamics simulations of a parallel (cis) homodimer were performed in a POPC lipid bilayer for 100 ns (Figure 15).

To shorten equilibration times, we simulated a dimer using HDOCK and then pre-oriented its best model (Figure S14, left) in a Golgi membrane using the Orientations of Proteins in Membranes (OPM) database (Figure S14, right). While docking models in water show a stable hydrophobic interface capable of shielding apolar residues (Figure S15), the lipid bilayer environment triggers immediate structural relaxation.

By 35 ns, the dimeric assembly exhibits clear signs of local instability, marked by a sharp increase in RMSD, partial unwinding of the core helical segments, and a systematic rearrangement of the monomers. Between 35 and 55 ns, a highly synchronized conformational transition occurs, resulting in a permanent loss of helicity and an increased interatomic distance between the two chains. This distorted state remains unchanged when extending the simulation to 200 ns.

This kinetic instability is driven by the polyanionic nature of ORF7b2. While the monomer possesses a narrow hydrophobic strip on one face, the remainder of its surface features a dense negative charge distribution. Lateral self-association of parallel monomers forces these highly charged patches into close proximity within the nonpolar bilayer core (dielectric constant approx. 2). This configuration creates severe electrostatic repulsion and steric clashes. To minimize this massive thermodynamic barrier, the helices undergo structural distortion and tilt toward the membrane-water interface to favor charge solvation. These findings demonstrate that parallel oligomerization is highly unfavorable within an apolar membrane environment, further challenging the sedentary transmembrane paradigm.

In summary, the simulation shows that the two components of the dimer change their relative orientation, as evidenced by an increase in RMSD, a decrease in overall helicity, and shifts in the monomers’ relative positions. Monomer distortion and partial unwinding reduce the overall alpha-helix content. Further experiments up to 200 ns show no significant changes. ORF7b2 exhibits a hydrophobic region confined to the same side of the molecule. The rest of the molecular surface exhibits a broad distribution of negative charge, which discourages interactions. If molecules interacted with the hydrophobic regions, the outer surface of the resulting system would be negatively charged, which would be unsuitable for an environment with a dielectric constant of approximately 2.

Structural characterization has highlighted that key chemical and physical properties determine the behavior of ORF7b2. In Figure S16, we show the different structural configurations of the dimer in the membrane at different times of the simulation. Electrostatic interactions are the driving force inducing structural deformations in the dimeric system.

### 2.5. Macromolecular Condensation Potential

#### Homotypic and Heterotypic Phase Diagrams

An interesting feature of ORF7b1 and ORF7b2 is their unexpected tendency to undergo liquid–liquid phase transitions. Indeed, some studies have shown that ORF7b2 interacts with viral proteins known to form droplets [93,94,95,96]. We conducted our analyses on the FINCHES web server at Washington University (St. Louis, MO, USA), which predicts IDR-mediated intermolecular interactions from sequence data.

**Figure 16:**
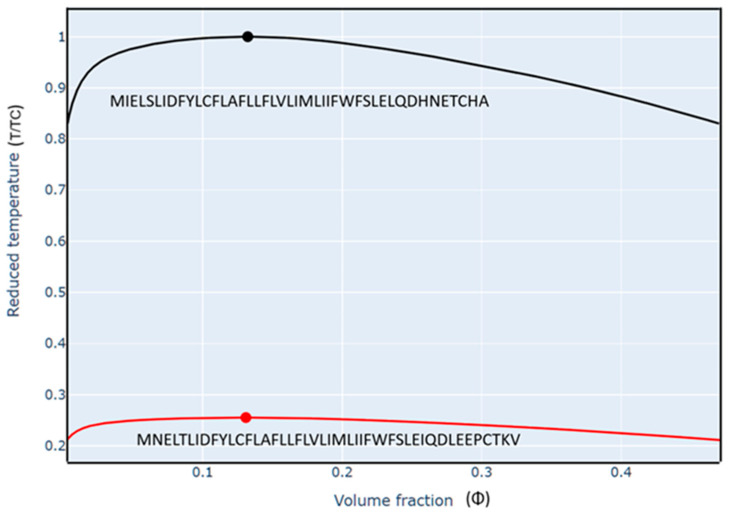
Phase diagrams of ORF7b1 (top) and ORF7b2 (bottom). The force field used to calculate the predicted phase diagrams was Mpipi-GG [115]. *X*-Axis Scale: linear. Critical points: in red for ORF7b1 and in black for ORF7b2. Lines in the diagram represent phase boundaries, where the protein transitions between phases (free protein and droplets). The reduced temperature is a normalized value, expressed relative to the critical temperature of the ORF7b2 sequence.

The presence of significant disordered fractions at the terminal tails prompted a qualitative prediction of intrinsically disordered region (IDR)-mediated liquid–liquid phase separation (LLPS) [97,98] using the Mpipi-GG force field (Figure 16). The calculated field parameters visually map the phase boundaries between free monomers in solution and condensed homotypic droplets as a function of normalized temperature (T/TC) and volume fraction (Φ).

The liquid–liquid phase diagram helps to understand the optimal stability range and operating conditions for intermolecular interactions. Therefore, evaluating protein phase diagrams is essential to understanding protein-environment interactions and their role in regulating function. However, with this approach, we can only qualitatively predict how differences in sequence will affect the relative diagrams [99,100].

The computed phase diagram demonstrates a massive thermodynamic divergence between the two orthologs. The area enclosed under the ORF7b2 phase boundary parabola is significantly larger than that of ORF7b1, revealing an exceptionally strong intrinsic propensity for homotypic droplet formation. Below the critical temperature point, ORF7b2 undergoes rapid condensation, shifting into a well-mixed single liquid phase only at elevated temperatures.

Phase diagrams help us understand how and when ORF7b1 and 2 respond to environmental changes [101,102]. The differences in protein behavior, although qualitative and potential, highlight that the conditions under which these proteins undergo liquid–liquid phase transitions are different [103,104]. The phase diagram of ORF7b2 suggests a higher intrinsic thermodynamic propensity, under simplified conditions, to form droplets. These equilibria are recognized as crucial for subcellular organization and several cellular functions [105]. However, we lack objective parametric evidence and precise, direct quantitative conditions for variables that definitively specify under which physiological conditions, or to what extent, the two proteins participate in cellular droplet formation through liquid–liquid phase separation in real cells.

While homotypic droplet formation in cellular environments is modulated by macromolecular crowding and post-translational modifications, verified interactomic data from BioGRID confirm that ORF7b2 physically targets highly multivalent viral partners known to act as potent droplet inducers [106,107,108,109].

Specifically, ORF7b2 binds the nucleoprotein (N) [106,107], which drives viral RNA condensation, as well as NSP3, ORF6, and numerous human cytosolic targets [108,109]. This expansive network of multivalent, reciprocal interactions strongly suggests that ORF7b2 functions in vivo as a dynamic modulator of biomolecular condensates [110,111]. This activity is vital for viral genome compartmentalization, replication efficiency [112], and host immune evasion. Other non-structural proteins, such as NSP3 and NSP12, can also interact with viral RNA to form biomolecular condensates [113]. One study suggests that the ORF6 protein also influences cellular compartmentalization and droplet formation [114]. According to another paper [10], several viral protein groups, including N, NSP3, ORF6, ORF8, ORF9b, and ORF7b2, interact with individual human proteins. The continuous, multiple, and reciprocal interactions between ORF7b2 and viral proteins involved in droplet formation in human cells suggest a potential role for ORF7b2 in the formation of biomolecular droplets via liquid–liquid phase separation.

### 2.6. Summary of Results

In synthesis, biophysical and structural profiling characterize ORF7b2 as a highly flexible, helical macromolecular polyanion adopting a prolate ellipsoidal geometry. At neutral pH, the protein maintains a strong net negative charge of −4 distributed across diffuse surface patches, combined with an asymmetric electric dipole vector tilted 24° outward from the core helix axis. This unique electrostatic topography renders the structure exquisitely responsive to minor fluctuations in ambient pH and ionic strength, promoting stable aqueous solubility specifically between pH 4.0 and 6.0 under low ionic strength conditions.

Consistent with these biophysical constraints, molecular dynamics simulations confirm that ORF7b2 resists parallel self-association within apolar bilayers, undergoing severe structural distortions due to the high thermodynamic cost of embedding uncompensated negative charges inside a hydrophobic core. Conversely, the protein exhibits a remarkable thermodynamic propensity to undergo liquid–liquid phase transitions and binds directly to key droplet-forming viral and host partners. Taken together, these computational findings shift the current structural paradigm, demonstrating that ORF7b2 operates as a dynamic, highly mobile peripheral membrane protein and a regulator of membraneless compartmentalization rather than a sedentary transmembrane component.

## 3. Discussion

The accessory protein ORF7b2 exhibits an unprecedented biophysical profile with no structural homologs outside its SARS-CoV ortholog, ORF7b1 [116,117]. A persistent misconception in structural biology assumes that because their central hydrophobic segments (residues 8–29) are identical, these two proteins must share identical cellular localizations and functions. This speculative view has led previous studies to overlook the terminal segments, despite their accounting for more than half of the total sequence. By integrating full-length 3D modeling, residue interaction networks, and molecular dynamics, our study establishes that while ORF7b1 behaves as a classic intrinsic membrane protein, ORF7b2 functions predominantly as a highly mobile peripheral factor or a dynamic soluble component.

The primary driver of this functional divergence lies in the contrasting electrostatic topography of the flanking tails. Unlike ORF7b1, which incorporates both positive and negative residues, the terminal segments of ORF7b2 are exclusively populated by negative charges. This uncompensated polyanionic density generates a severe asymmetric surface charge distribution at physiological pH [118]. It also imposes a steep pH-dependent titration profile that renders the structural architecture extraordinarily responsive to minor environmental fluctuations, contrasting with the narrow, stable electrostatic plateau observed for ORF7b1. However, other factors, such as surface charge, hydrophobicity, and structural flexibility or rigidity, influence protein-polyanion interactions [119,120].

This thermodynamic and stereochemical asymmetry directly explains the kinetic instability of ORF7b2 inside lipid bilayers [121,122]. Free energy evaluations of cytoplasmic-to-membrane translocation—calculated via the Hessa hydrophobicity scale [123,124], yield a highly unfavorable positive net transfer energy (ΔG_transfer_ = +3.49 kcal/mol). This barrier confirms that a sedentary hydrophobic membrane environment is thermodynamically hostile to the uncompensated polyanionic sequence of ORF7b2.

Furthermore, membrane molecular dynamics simulations demonstrate that parallel (*cis*) homodimers undergo immediate structural relaxation and helix unwinding. The outward 24° tilt of the macro-dipole vector and the steric/electrostatic clashes generated by forcing dense negative patches into the low-dielectric core (approx. 2) prevent stable transmembrane self-association. To minimize this energetic penalty, the protein undergoes mechanical distortions, forcing its segments toward the membrane-water interface where electrostatic attraction dominates.

This pronounced structural plasticity and environmental responsiveness are finely coordinated by specific intramolecular hinges. Normal Mode Analysis maps long-range backbone collective motions directly onto the topological hinges (Phe9, Leu20, and Leu32) identified via Residue Interaction Networks (RINs). While the compact, short rigid core of ORF7b2 undergoes restricted bending, its extensive non-helical terminal tails experience continuous, large-scale fluctuations spanning up to 30 Å. This highly entropic, flexible ensemble prevents rigid folding and aggregation, maintaining a compact prolate ellipsoidal geometry in solution.

Crucially, bioinformatic screening confirms that ORF7b2 lacks the canonical N-terminal signal peptides required for co-translational translocon-mediated entry into the endoplasmic reticulum (ER) or Golgi membranes [125,126,127]. Instead, post-translational screening identifies a specific, highly conserved Hsp70-chaperone binding heptad at positions 24–30 (Figure S17). This potential structural motif implies an alternative, unconventional post-translational trafficking pathway, where cytoplasmic molecular chaperones actively preserve monomer solubility and direct alternative subcellular localization [127,128,129,130].

Rather than executing a sedentary structural role inside lipid bilayers, the biophysical traits of ORF7b2 favor a dynamic participation in liquid–liquid phase separation (LLPS). The highly expanded homotypic phase diagram of ORF7b2 reflects an intrinsic thermodynamic propensity to undergo condensation and compartmentalization into membraneless droplets. This predictive capacity aligns precisely with established heterotypic interactomic data from BioGRID and cell-based assays. By physically targeting well-known viral droplet inducers, ORF7b2 may exert critical regulatory controls during infection:

(1) **Viral Genome Organization**: Direct binding to the multivalent nucleoprotein (N) can stabilize host-free macromolecular condensates, directly modulating viral RNA packaging and replication efficiency [111,112,113].
(2) **Complex Assembly Regulation**: Interactions with the poly-ADP-ribose (PAR) domain of NSP3 inside multi-protein complexes suggest a role in coordinating the kinetics of viral replication factories.
(3) **Host Immune Evasion**: The incorporation of host cytosolic or membrane-associated factors into these dynamic biomolecular droplets can selectively block intracellular trafficking or suppress host antiviral signaling pathways, promoting viral persistence [113,114,115].
(4) **Therapeutic Targeting**: Since IDR-mediated condensate formation is essential for the viral life cycle, disrupting the specific pH-responsive phase boundaries or targeting the core hinge segments of ORF7b2 represents a viable pharmacological strategy to halt viral assembly.

While this study relies on convergent computational methods (ab initio modeling, RIN, NMA, and MD), the high consistency of these independent biophysical parameters serves a critical screening purpose. Rather than substituting experimental biology, our structural and thermodynamic profiling defines the exact molecular boundaries that explain why categorizing ORF7b2 solely as a transmembrane protein is reductive. These findings provide the essential structural framework to guide and rationalize upcoming biochemical, biophysical and cell-based assays. Overall, ORF7b1 displays characteristics of an intrinsic membrane protein, while ORF7b2 more closely resembles a peripheral membrane protein, matching most of the specific features discussed in Appendix A.

## 4. Methods

### 4.1. Electrostatic Properties

The charge distribution of proteins was evaluated according to Das and Pappu [41,42,43,131]. We calculated the fraction of charged residues as FCR = |f+ + f−| and the net charge per residue as NCPR = |f+ − f−|. Here, f+ and f− represent the fractions of positive and negative charges, respectively. These values help classify protein sequences into distinct regions of the IDP Diagram of States [43]: (i) weak polyampholytes and polyelectrolytes, called Region 1, with FCR < 0.25 and NCPR < 0.25, showing a tendency for Globule and Tadpole ensembles; (ii) the boundary region or Region 2 between 1 and 3, characterized by 0.25 ≤ FCR ≤ 0.35 and NCPR ≤ 0.35; (iii) strong polyampholytes (Region 3) with FCR > 0.35 and NCPR ≤ 0.35, favoring Coil, Hairpin, and Chimera ensembles; and (iv) strong polyelectrolytes (Region 4), where FCR > 0.35 and NCPR > 0.35, with a propensity for Swollen Coil ensembles. Finally, we calculated the parameter k to differentiate sequence variants based on the linear distribution of oppositely charged residues [41]. The overall charge asymmetry was computed as σ = (f+ − f−)**^2^**/(f+ + f−). For each sequence variant, k was determined by dividing the sequence into N overlapping segments of size g. For each segment, we calculated σi = (f+ − f−)**^2^**i/(f+ + f−)**^i^**, representing the charge asymmetry of that segment. We quantified the squared deviation from σ as

$$\delta =\left[\sum_{j=1\to Nblob}{\left(\sigma i\;-\;\sigma \right)}_{2}\right]/Nblob$$

We used g (group of residues, or blob) = 5 and hypothesized various sequence variants, evaluating different values of δ for each variant. Hence, the maximal value δmax for an amino acid composition was used to define k = (δ/δmax).

### 4.2. Net Charge Calculation

The net charges of proteins at a given pH are based on the formula below:

Z = ∑i Ni [10pKai/(10pH + 10pKai)] − ∑j Nj [10pH/(10pH + 10pKaj)]

where Z is the net charge of the peptide sequence. Ni: number of arginine, lysine, and histidine residues, including the N-terminus; pKa values of the N-terminus and these residues; Nj: number of aspartic and glutamic acids, cysteine, and tyrosine residues. The pKa values for these amino acids are: cysteine (8.33), aspartic acid (3.86), glutamic acid (4.25), histidine (6.0), lysine (10.53), arginine (12.48), tyrosine (10.07), the N-terminal (9.69), and the C-terminal (2.34). Also, the data includes the pH and pKa values for these residues and the C-terminus. The isoelectric point is the pH at which peptide Z has a net charge of zero. Biochemistry textbooks provide formulas and pKa values.

### 4.3. Dipole Moment

The dipole moment, in Debyes, is the magnitude of the dipole vector D = 4.803 × Σriqi, summed over all atoms ‘i’, where 4.803 converts from Angstrom-electron-charge units to Debyes. We calculated the mass moment vector of the protein as Rx = Σxi^2^, Ry = Σyi^2^, and Rz = Σzi^2^, and the associated mean radius RM = [(Rx + Ry + Rz)/3]**^1/2^** measures the overall protein size. We also used the Protein Dipole Moment Server [90] at http://bip.weizmann.ac.il/dipol (accessed on 28 June 2026) for the calculations.

### 4.4. CIDER (Classification of Intrinsically Disordered Ensemble Regions)

CIDER (Classification of Intrinsically Disordered Ensemble Regions) is a web server developed by the Pappu lab [43] at Washington University in St. Louis. CIDER facilitates the calculation of many parameters related to any protein sequence. It is especially accurate for small proteins. The server is available at http://pappulab.wustl.edu/CIDER/analysis/ (accessed on 28 June 2026). The calculation of a peptide’s average hydrophilicity is based on data from Hopp & Woods [132]. As the authors clearly explain, CIDER does not calculate intrinsic disorder but accounts for it in its calculations of small protein conformations, where present.

### 4.5. Phase Diagram

We created the diagrams on the FINCHES web server (https://www.finches-online.com/ accessed on 28 June 2026), a Python package at Washington University in St. Louis, USA. It predicts IDR-mediated intermolecular interactions using only sequences. The calculations were carried out, as shown by Ginell, G. et al. [115] and Garrett, M. et al. [133]. The platform offers a bottom-up approach that uses chemical physics derived from coarse-grained force fields to predict IDR-mediated interactions. This approach assumes that the amino acid sequence alone (considering local sequence context) captures the chemical specificity of IDRs, and that local attractive and repulsive interactions can be predicted and used to identify subregions within an IDR that may facilitate such interactions. This enables quick and verifiable predictions of which protein regions and residues are likely to interact with a binding partner. Using this approach, we predicted phase diagrams and qualitatively assessed how sequence changes should influence them.

One application of this method is predicting phase diagrams between two homologous proteins directly from their sequences. The predictions are based on parameters obtained from coarse-grained molecular mechanics force fields. We employed the Mpipi-GG-based (V1) force field to generate these diagrams [102,134]. These predictions (at least qualitatively) highlight how sequence chemistry influences phase behavior and clarify how sequence modifications impact intermolecular interactions during IDR-mediated phase separation. We construct the predicted phase diagrams by first calculating the overall mean-field homotypic interaction parameter, converting it into a Flory-Chi parameter, and solving the phase diagram using the analytical approach developed by Qian, Michaels, and Knowles [135]. Comparing two sequences with mutations is the most effective way to assess how these mutations influence phase behavior. It is important to note that these phase diagrams provide a qualitative, not quantitative, description of phase behavior and phase boundary predictions. Several considerations are crucial when interpreting these diagrams. This report presents phase-diagram temperatures versus volume fraction, with temperature reduced. This reduced temperature is normalized to the ORF7b2 sequence’s critical temperature. Therefore, the absolute value of the reduced temperature is meaningful only when comparing ORF7b1 and ORF7b2 sequences. Understanding a sequence’s phase behavior allows us to predict whether another sequence will behave similarly or differently. However, this comparison is only relative since we lack data to quantify these behaviors in absolute terms. To assess disorder across the two sequences, we used Metapredict version 3, a deep-learning-based consensus predictor of intrinsic disorder and predicted structure [115,136]. It generates a high-resolution, interactive plot of per-residue disorder and the predicted AlphaFold2 structural confidence score.

### 4.6. Structure Modeling

PHYRE2, Protein Homology/AnalogY Recognition Engine V 2.0 is a web-portal for protein modeling, prediction, and analysis [55,137] at the Structural Bioinformatics Group, Imperial College, London, UK (http://www.sbg.bio.ic.ac.uk/~phyre2/html/page.cgi?id=index accessed on 28 June 2026). Phyre can detect remote homology to known structures significantly beyond the range of the popular PSI-Blast. Advanced profile-profile matching techniques, loop modeling, and side-chain placement algorithms enable the construction of accurate full-atom models based on homology to known protein structures, even with sequence identities < 15%. PEP-FOLD3 is a de novo method for predicting peptide structures from amino acid sequences, based on 100 simulations [56,57,58]. Each simulation explores a different region of the conformational space (it limits predictions to amino acid sequences between 5 and 50 residues in FASTA format). It returns an archive of all the generated models, including the clusters’ details and the best conformation of the top 5 clusters. Once completed, a Monte Carlo procedure refines the peptide structure. (https://bioserv.rpbs.univ-paris-diderot.fr/services/PEP-FOLD3/ accessed on 28 June 2026) MEMEMBED 1.15 (Bio-informatics Group–University College London) Membrane Protein Orientation Predictor (https://mybiosoftware.com/memembed-1-15-membrane-protein-orientation-predictor.html accessed on 28 June 2026) accurately orients and refines both alpha-helical and beta-barrel membrane proteins within the lipid bilayer using a genetic algorithm and knowledge-based statistical potential [138]. The Workbench offers a variety of protein structure prediction methods. The site can be used interactively via a web browser or programmatically via our REST API.

### 4.7. HINGEProt

HINGEProt (http://bioinfo3d.cs.tau.ac.il/HingeProt/hingeprot.html accessed on 28 June 2026) is a web server for Protein Hinge Prediction Using Elastic Network Models [84]. HingeProt uses both the Gaussian Network Model (GNM) [139,140] and the Anisotropic Network Model (ANM) [83]. GNM analyzes the fluctuations of N residues in a structure by decomposing them into N-1 nonzero modes, based on the Cartesian coordinates of Ca atoms. It identifies the eigenvectors associated with the slowest first and second modes. The square of these vectors indicates the mean-square fluctuations (autocorrelations) of residues from their equilibrium positions along the primary modes. Minima of these fluctuations at a mode mark flexible joints in the structure, i.e., hinge regions connecting rigid units and mobile loops. These hinge regions are mechanistically significant, mediating cooperative motions of functional importance. GNM calculates the mean-square fluctuations and the correlations between residue fluctuations in the two slowest (most dominant) modes, which correspond to protein motions. This reveals hinge regions and their cooperative behavior. ANM describes the direction of fluctuations in these modes, since GNM fluctuations are isotropic. It predicts the fluctuations of N residues in the x, y, and z directions around the average structure (from X-ray or NMR data) in 3N-6 nonzero modes [83]. ANM analysis specifies the displacement directions of residues in GNM’s two slowest modes by matching ANM modes to GNM modes by comparing squared fluctuations. Since equilibrium positions fluctuate symmetrically, we can create ANM-predicted deformed structures by adding or subtracting these fluctuations from each residue’s equilibrium position.

### 4.8. Molecular Dynamics

The GROMACS software (v4.5.6) performed molecular dynamics (MD) simulations [141,142] on the best model of ORF7b2 using the GROMOS43a1 all-atom force field at neutral pH. We conducted each simulation three times, and the results were consistently similar. In a previous paper of ours [143], we evaluated this force field and found it to be among the most suitable for simulating the folding of short peptides. We placed the model in a cubic box with 86.2 Å sides and solvated it with 21,329 SPC216 water molecules. Initially, we performed 2000 steps of energy minimization and 25,000 steps of position restraints to equilibrate the protein and balance the surrounding water molecules. We subjected the complete 3D structure of ORF7b2 to MD simulations for 40 ns in explicit water, with a time step of 2 fs, a temperature of 300 K, a time constant of 0.1 ps, and pH 7.0. We performed a second set of experiments in a solvated lipid bilayer under similar experimental conditions with a dimeric 3D structure of ORF7b2 present. HDOCK modeled the structure. To achieve this, we integrated a pre-oriented dimeric ORF7b2 model from the OPM database (http://opm.phar.umich.edu accessed on 28 June 2026) into a 130-POPC lipid bilayer, built with VMD’s membrane builder, taking into account its residue hydrophobicity. This approach rigorously calculates, based on energetic and thermodynamic considerations, how the helix embeds in the membrane. The OPM model is shown in the Figure S14. After inserting the correctly oriented helix into the membrane, we solvated the entire system in a box containing 10,985 water molecules. Subsequently, we used VMD to ionize the system and processed it through three steps: (i) equilibration and melting of lipid tails, (ii) minimization and equilibration with the protein constrained, and (iii) equilibration with the protein released. After these three steps, we subjected the entire system to MD simulation for 100 ns, at 300 K and neutral pH.

### 4.9. Molecular Dynamics Analysis

We analyzed the trajectories, which contain information about the time evolution of all the atoms’ coordinates, using various GROMACS routine utilities. These utilities include root-mean-square deviation (RMSD), gyration radius (Rg), root-mean-square fluctuations (RMSF), helicity, total solvent accessible area (ASA), and others. Principal Components Analysis (PCA) was used to calculate the relevant functional motions. We calculated the number of H-bonds and interactions with their closest atoms (IAC) using the Protein Interactions Calculator (PIC), HBPLUS, and COCOMAPS tools.

### 4.10. (ORF7b2-ORF7b2) Docking

HDOCK server (http://hdock.phys.hust.edu.cn/ accessed on 28 June 2026), a web server for protein-protein docking based on a hybrid strategy [144], was used to model ORF7b2 dimerization in silico. The information entered for receptor and ligand molecules was the best ORF7b2 Phyre2 model. The server automatically predicts their interaction through a hybrid algorithm of template-based and template-free docking. Data input that accepts sequence and structure is the first step of the process. The second step of the workflow is a sequence similarity search. The workflow uses the input sequences, or those converted from structures, to conduct a sequence similarity search against the PDB sequence database. This search identifies homologous sequences for both receptors and ligand molecules. In the third step, we compare PDB codes and select a common template for receptors and ligands. If the two sets of homologous templates show no overlap, we will select the best template for the receptor protein and/or the ligand protein from each set. If multiple templates are available, we select the one with the highest sequence coverage, sequence similarity, and resolution. Using the selected templates, MODELLER builds models; ClustalW conducts the sequence alignment. The last step is traditional global docking. Here, HDOCK-lite, a hierarchical FFT-based docking program, is used to sample putative binding orientations. A web page interactively displays the top 10 docking models.

### 4.11. Orientations of Proteins in Membranes (OPM) Database

OPM provides spatial arrangements of membrane proteins regarding the core of the lipid bilayer [145]. OPM offers preliminary results from a computational analysis of transmembrane α-helix binding in experimental structures for dimeric proteins. The PPM3 server positions proteins within a bilayer of adjustable thickness and curvature to minimize their transfer energy from water to the membrane. The server treats each protein as a rigid body floating in a hydrophobic slab of variable thickness. Our experiment used a membrane with a Golgi-like composition, 29.4 ± 2.7 Å thick. The orientation of the proteins was determined by minimizing their overall transfer energy to –28.8 kcal/mol, considering variables in a coordinate system with the *Z*-axis coinciding with the bilayer normal. We calculated the longitudinal axes of TM proteins as the average of TM segment vectors. The resulting tilt angles were 13 ± 2° and 15 ± 2.5° for the two monomers. To pre-orient probable transmembrane proteins in a lipid sheet system, we used the OPM server. This method reduces equilibration times in membrane molecular dynamics simulations. We present the orientation results in Figure S14.

### 4.12. Charge Distributions and Electrostatic Potential Calculations (http://compbio.clemson.edu/delphi_webserver Accessed on 28 June 2026)

DelPhi calculated charge distributions and electrostatic potentials using a finite-difference solution of the Poisson–Boltzmann equation [92]. DelPhi is an electrostatics simulation program that can analyze electrostatic fields in various molecular systems, including proteins [146]. It accepts a coordinate file as input. DelPhi includes solutions to the nonlinear Poisson–Boltzmann equation, offering more accurate results for highly charged protein systems. Many features enhance Delphi’s speed and versatility, enabling it to handle complex systems and high-dimensional finite-difference lattices. We ran the DelPhi executable on a server equipped with Fortran and C compilers. The program can be downloaded from: https://honiglab.c2b2.columbia.edu/software/cgi-bin/software.pl?input=DelPhi (accessed on 28 June 2026) at Columbia University. The input PDB file should be in PQR format, which includes atomic radii and charges. We used PDB2PQR [147], a Python software package, for this purpose. This package automates routine tasks in preparing structures for continuum electrostatics calculations and provides a platform-independent utility for converting PDB protein files to PQR format. An analysis is required to read and display the potentials and to analyze the results. The program also allows users to output a potential map, which they can visualize and contour in PyMOL (or even Biosym). A utility is available to facilitate this.

### 4.13. Effect of pH on Protein Stability

We used Protein-Sol, a web server operated by the University of Manchester, UK (https://protein-sol.manchester.ac.uk/ accessed on 28 June 2026), dedicated to calculating scaled solubility values and various stability parameters (heat maps) for proteins [49,50]. The server uses both sequence data and 3D models for its calculations. The Protein-Sol sequence algorithm calculates 35 features related to protein solubility, including folding propensity [148], disorder propensity [149], beta strand propensities [150], Kyte-Doolittle hydropathy [44], pI, sequence entropy, and absolute charge at pH 7. it assesses the solvent accessible surface area (SASA) for each atom (considered buried if SASA < 5Å**^2^** and sur-face-accessible otherwise), as well as the ratio of non-polar to polar (NPP ratio) values at the interface, which informs the predicted sign of the net charge per residue. This information is used to generate heat maps depicting the dependence of protein stability in the folded state on pH and ionic strength, using the Debye-Hückel (DH) method for interactions between ionizable groups and pKa calculations. The heat maps display the predicted net charge (in electrostatic units per amino acid) and the pH-dependent contribution to stability (in Joules per amino acid). Further details are available in the articles [149,150].

### 4.14. Residue Interaction Network Generator 

Residue Interaction Network Generator (RING 4.0: https://ring.biocomputingup.it/ accessed on 28 June 2026) is an online platform to calculate graphs (interactomes) of residue-residue interactions of single proteins by a web server called “Residue Interaction Network Generator 4.” It analyzes how different parts of molecules (especially proteins) interact with each other [73]. Node representation—Closest (default): The system considers all atoms of the residue (or group) when measuring the distance. This option is convenient for PDBs with a safe resolution when considering side-chain coordinates. The program always processes ligands or hetero groups with all atoms.

Edge representation (cardinality): The RING algorithm identifies all interactions that connect chemical components. The Chemical Component Dictionary (PDB HET dictionary), an external reference file describing all residue and small-molecule components found in PDB entries, defines them. The hydrogen bond’s maximum donor-acceptor distance was 3–9 Å, with an angle ε > 90° [151], while the H-acceptor distance was 2.5 Å for h-bonds, 6.5 Å between aromatic ring centers for π-π interactions [152], and 0.01 Å for the intersection between two atoms’ van der Waals radii (0.0–1.0). RING can estimate the vdW interactions. Unless otherwise specified, we calculate the distance between a pair of atoms using their centers (i.e., the 3D coordinates in the PDB file).

### 4.15. Centrality Analysis

The graphs are available as downloadable JSON files for use in Cytoscape. Cytoscape performed the Centrality analysis. It identifies a network’s most central, or significant nodes. A single index does not define centrality; rather, several indices capture different structural aspects of the interactions a researcher may focus on. Residues crucial for 3D folding or function are high-centrality nodes [153]. Edge betweenness is a vital edge centrality measure that shows the topological importance of edges in the network. Specifically, it is linked to interactions between two parts of a structure, such as domain boundaries and interfaces in multimeric proteins and protein complexes, enabling inter-domain and protein–protein interactions. RING3 is a tool that can analyze how interactions within a molecule change when it changes shape. It takes structural data from PDB files, even when those files represent multiple molecule versions. Closeness Centrality is a network measure of nodal importance, quantifying how prominent a node is relative to others [154]. Closeness centrality (Ci) assesses the proximity of node i to all other nodes within the network. Statistically significant central residues are evaluated using the z-score values of the residue closeness centrality, defined by Zk = Ck − C¯/σ, where Ck is the closeness centrality of residue k, C is the mean closeness centrality value, and σ is the corresponding standard deviation [154]. Residue centrality can identify the protein core and peripheral residues of membrane proteins [155].

## 5. Conclusions

Rather than acting as a sedentary, structural transmembrane anchor confined strictly to the Golgi apparatus or endoplasmic reticulum, our computational profiling demonstrates that SARS-CoV-2 ORF7b2 operates as a dynamic, highly mobile peripheral membrane protein and a flexible regulator of host cell biology. While integral transmembrane proteins are rigidly locked within lipid bilayers, the uncompensated polyanionic surface charge and outward-tilted helix macrodipole of ORF7b2 generate a steep thermodynamic barrier (ΔG_transfer_ = +3.49 kcal/mol) for sedentary membrane insertion. This biophysical constraint promotes rapid, pH-dependent detachment, allowing the monomeric pool to partition between the membrane–water interface and the aqueous cytosolic environment.

This spatial plasticity is finely governed by a unique combination of structural and sequence-encoded features. The central helical core exhibits a low hydrophobicity x volume (HV) product (Figure S18) and is split by specific topological breakpoints, facilitating low-frequency bending and twisting motions [156]. Flanked by highly mobile, intrinsically disordered, and exclusively negative terminal tails, this entropic ensemble permits extensive backbone fluctuations of up to 30 Å without structural unfolding.

The resulting dynamic surface topography allows ORF7b2 to overcome the spatial restrictions of a single organelle docking site. Experimentally validated interactomic data from BioGRID confirm that ORF7b2 targets an expansive network of physical partners distributed across diverse host cellular compartments, directly modulating immune responses, intracellular signaling, and viral replication [8]. Crucially, the expanded phase boundary architecture of ORF7b2 dictates an intrinsic propensity to undergo liquid–liquid phase separation, enabling it to act as a crucial structural modulator of host-free biomolecular condensates via direct heterotypic recruitment of multivalent viral factors like the nucleoprotein (N) and NSP3.

In summary, this study establishes a robust physicochemical and structural predictive framework that resolves previous interpretative uncertainty regarding the subcellular localization and functional deployment of ORF7b2. By mapping the precise stereochemical boundaries, pH-sensitive transition zones, and central hinge residues of this unique viral factor, integrating sequence-based modeling, structural prediction, and thermodynamic stability analyses, our work delineates a set of testable molecular mechanisms governing its membrane association, conformational switching, and potential interaction interfaces with host or viral partners. These integrated profiles now define the key molecular hypotheses that should be prioritized in future in vitro and in vivo cellular assays (e.g., pull-down assays to identify direct binding partners, surface plasmon resonance to quantify binding affinities and kinetics, or cell-based confocal and super-resolution microscopy to track condensate or droplet formation and subcellular relocalization over time) to resolve the spatial, temporal, and functional dynamics of this peculiar protein throughout the viral replication cycle and host-cell infection process.

## Figures and Tables

**Table 1:** Charge distribution analysis of ORF7b1 and ORF7b2.

Physical-Chemical Parameters	ORF7b1	ORF7b2	Notes
N (MW)	44 (Mw 5301.51)	43 (Mw 5179.31)	Fraction of negative residues
f−	0.13636	0.11628	Fraction of negative residues
f+	0.02273	0.00000	Fraction of positive residues
FCR	0.15909	0.11628	Fraction of charged residues
NCPR	−0.11364	−0.11628	Net charge per residue
Sigma	0.08117	0.11628	Charge asymmetry
Delta	0.03182	0.01706	Square deviation of every blob σ value from the sequence’s mean σ value
Max Delta	0.08945	0.06725	δ value associated with the segregated sequence of the charge composition provided
pI	3.72	4.32	Isoelectric point at pH 7.00
AH	−0.83	−0.98	Average hydrophilicity
Phase Plot (Region)	1	1	(See the state diagram)
Phase Plot Annotation	Globule/Tadpole	Globule/Tadpole	Prolate elongated structures
Polymeric State	Weak negative polyampholyte	Weak negative polyampholyte	

Note: We evaluated the protein’s charge distribution using the methods of Das and Pappu [41,42], calculating the fraction of charged residues (FCR) as FCR = |f+ + f−| and the net charge per residue (NCPR) as |f+ − f−|. In this context, f+ and f− denote the fractions of positive and negative charge, respectively. Sigma, σ = [f+ − f−]/[f+ + f−], where f− and f+ refer to the fractions of negative and positive residues across the entire sequence, and σ indicates their distribution symmetry. These values enable the classification of the behavior of segmental protein sequences into distinct regions of the Diagram of States for IDPs. We calculated the PI according to Lukasz et al. [43] and the AH according to Kyte and Doolittle [44].

**Table 2:** ORF7b2 hinge residues.

The Slowest Mode 1			
Rigid Part No	Residues	Score	Hinge Residues
1	1–20	0.88	20–21
2	21–43	0.9	20–21
**The Slowest Mode 2**			
1	1–9	0.68	9–10
2	10–32	0.82	32–33
3	33–43	0.85	32–33

Note: The table shows the best hinge residues in the ORF7-b2 structure and the reliability of the result as calculated by HINGE-Prot [the score varies between 0 and 1]. These residues define twist angles or rigidity points that organize the entire structure’s movement (see also Figure 10).

## Data Availability

Data is contained within the article or Supplementary Material.

## Supplementary Materials

The following supporting information can be downloaded at https://www.mdpi.com/article/10.3390/ijms27136022/s1.

## Appendix A

ORF7b2 and peripheral and monotopic membrane proteins.

Understanding the difference between peripheral and monotopic membrane proteins is essential for classifying ORF7b2; this study, for the first time, provides the structural and physical-chemical data necessary for this classification. It is also interesting to consider how ionic strength influences the function of membrane-bound proteins in different environments.

The ionic strength near the membrane surface.

Under normal physiological conditions, no cell compartment has an ionic strength close to zero. Even seemingly “empty” or isolated compartments contain dissolved ions to maintain osmotic balance and membrane potential. However, newly formed endocytic vesicles or artificial vesicles (liposomes) in the laboratory may initially have a very low ionic strength, but this is not a stable state in vivo [157]. When a vesicle forms through mem-brane invagination, it initially contains dilute extracellular fluid; its lower ionic strength than the cytoplasm generates phase droplets of low ionic strength. Specific cellular stresses can also cause transient dilution of cytoplasmic contents, temporarily altering ionic strength. Some proteins, such as environmental sensors, can turn on or off in response to changes in ionic strength. Inter-membrane spaces (such as between mitochondrial membranes) can also have a transient ionic composition, but not zero. However, we can subtly and interestingly extend the concept of ionic strength in biological membranes. Lipid membranes are hydrophobic barriers; they do not contain free water or dissolved ions. Therefore, we cannot speak of ionic strength within the lipid bilayer. Ionic environments immerse the membrane surfaces, placing the membrane’s two faces (inner and outer) in contact with aqueous solutions (cytoplasm and extracellular or luminal space). These environments have a well-defined ionic strength that directly influences the distribution of charges on the membrane surface and the interaction with peripheral proteins or cytoplasmic domains of integral proteins. Near the membrane surface, ionic microenvironments generate a layer of counterbalancing ions, a Debye layer (e.g., positive ions near negative lipid phosphates). This creates an ionic microenvironment with characteristics different from the bulk solution, with a local ionic strength that affects interactions with charged proteins [158,159].

Membrane peripheral proteins.

Peripheral and monotopic membrane proteins generally consist of two parts: a non-polar helix, which is partially embedded in the membrane or interacts with the surface via hydrophobic forces, and a flexible, disordered, negatively charged tail often involved in regulation or molecular interactions. Local variations in ionic strength and pH continually influence peripheral membrane proteins, and this sensitivity plays a crucial role in their biological functions [160]. Changes in ionic strength can activate or deactivate these proteins, such as when they serve as environmental sensors. Many peripheral proteins characterized by a hydrophobic helical domain and a negatively charged, flexible tail can operate in different cellular compartments and orchestrate various metabolic processes, thanks to their structural modularity and responsiveness to the local biochemical environment [161,162]. While monotopic proteins are stably anchored to the membrane, ionic strength can impact their outer domains. An example includes GPI-anchored proteins, monotopic proteins attached to the cell membrane via glycosylphosphatidylinositol (GPI) anchors [163].

Cell signaling is intertwined with its functional significance, as many signaling proteins transiently associate with the membrane in response to ionic shifts, such as in vesicular trafficking, with BAR or ENTH proteins binding only under favorable ionic conditions [164]. Complex assembly, like the formation of signal platforms such as synapses or immunological synapses, can also be regulated by ionic forces. Concrete examples of peripheral proteins with a bipartite architecture include:

1. MARCKS (Myristoylated Alanine-Rich C Kinase Substrate), which features a hydrophobic domain and a flexible, negatively charged tail rich in glutamate and aspartate that interacts with PIP_2_ and other proteins [165]. It influences the cytoskeleton, signaling pathways, and vesicular trafficking, with ionic strength and phosphorylation modulating its membrane association. Elevated Ca^2+^ levels promote binding to phospholipids in a pH- and ion-dependent manner, while reduced ionic strength enhances membrane association through charged interactions.
2. Amphiphysin/Endophilin (BAR proteins), which possess a banana-shaped structure with a hydrophobic surface that associates with membranes and induces curvature [166]. Their disordered tails contain negatively charged regions and motifs for binding other proteins, playing roles in vesicle fission and membrane shaping.
3. The α-synuclein family proteins, which are peripheral proteins. The N-terminal domain forms an amphipathic helix that interacts with membranes [167], while the C-terminal tail is intrinsically disordered and negatively charged, facilitating protein–protein interactions to regulate synaptic trafficking [168].
4. Annexins [169], which bind phospholipids in a pH- and ion-dependent manner, especially in the presence of increased Ca^2+^. The functional traits of these charged tails hinge on their flexibility, allowing for transient and adaptable interactions. Conversely, their negative charges favor interactions with positively charged proteins or ions such as Ca^2+^.

Environments with low ionic strength do not shield electrostatic interactions between charged groups, increasing attraction or repulsion between charged domains. Excessive surface charges can destabilize proteins, leading to conformational changes or aggregation. Numerous complexes, such as ribosomes, spliceosomes, and transcription complex-es, depend on optimal ionic strength to maintain their structure. Low ionic strength environments may cause disassembly or alter interaction strength depending on charge distribution. Therefore, shifts in ionic strength and pH are vital for regulating mem-brane-protein associations because peripheral proteins do not remain permanently attached to the membrane. Changes in these conditions can promote or inhibit binding to membrane lipids or trigger conformational shifts that activate or deactivate protein functions.

Cells consciously localize peripheral proteins within microenvironments characterized by different pH or ionic strength [170]. For example, during endocytosis, vesicles form in locally acidic or calcium-rich environments. In synaptosomes, rapid ionic changes control signaling protein activation. Physiologically, this allows cells to turn signaling pathways on or off by adjusting local pH or ionic strength. Vesicular trafficking activates proteins only within specific pH conditions (such as in endosomes), and stress responses, like osmotic shocks or ionic shifts, can free peripheral proteins from membranes, altering cellular responses.

In summary, peripheral proteins operate precisely because they can respond to local changes in ionic strength and pH. This makes them versatile and adaptable tools for cell regulation. We documented a well-defined bipartite organization for ORF7b2, but we did not examine ORF7b1 at the same level of detail, as our primary goal was to highlight its structural and functional differences from ORF7b2, where appropriate.
