# Supplementary material for: Structural Features of a Tiny Viral Protein, ORF7b of SARS-CoV-2
Source: Int J Mol Sci. 2026 Jul 4;27(13):6022. doi: 10.3390/ijms27136022 (PMC13362368; doi:10.3390/ijms27136022)
Supplement: Supplementary file 1 [file ijms-27-06022-s001.zip › ijms-4366328-supplementary.pdf]

Supplements to the article:

Structural Features of a Tiny Viral Protein, ORF7b of SARS-CoV-2

Giovanni Colonna

Medical Informatics Unit, Azienda Ospedaliera Universitaria Luigi Vanvitelli,
Università degli Studi della Campania, 80138 Naples, Italy;
giovanni.colonna@unicampania.it

Other experimental results:

Table S1 Comparison between ORF7b1 and ORF7b2 interactions as curated by BioGRID

Interacting Protein	Biological Entity of Belonging	Interaction
E, ORF3A, ORF3B, ORF6, ORF7A, ORF7B, ORF8A, ORF8B, ORF9B, ORF14, NSP9AB, NSP16B.	SARS-CoV1	Physical (LT): ORF7b1-interactor
E, N, M, ORF7b, ORF7A, ORF6, ORF3A, NSP8, NSP7, NSP4, NSP2, NSP1.	SARS-CoV2	Physical (LT): ORF7b2-interactor
EC61A1, HLADRA, HSP90AB1, HSP90AA1, IFITM1, MAVS, OSBP, STT3A, TNFAIP2.	Homo sapiens	Physical (LT): ORF7b2-interactor

Note: The BioGRID Project highlights and curates interactions found in human cell models in vitro.

Table S2 - Amino acid composition

	ORF7b2*		ORF7b1**	
Amino acid	Number of residues	Percentage %	Number of residues	Percentage %
Ala [A]	2	4.7	1	2.3
Asn [N]	1	2.3	1	2.3
Asp [D]	2	4.7	2	4.5
Cys [C]	2	4.7	2	4.5
Gln [Q]	1	2.3	1	2.3
Glu [E]	3	7.0	4	9.1
His [H]	2	4.7	-	-
Ile [I]	5	11.6	5	11.4
Leu [L]	11	25.6	11	25.0
Lys [K]	-	-	1	2.3
Met [M]	2	4.7	2	4.5
Phe [F]	6	14.0	6	13.6
Pro [P]	-	-	1	2.3
Ser [S]	2	4.7	1	2.3
Thr [T]	1	2.3	2	4.5
Trp [W]	1	2.3	1	2.3
Tyr [Y]	1	2.3	1	2.3
Val [V]	1	2.3	2	4.5

Note: Negative residues are in red; positive residues are in blue.
*Total number of negatively charged residues (Asp + Glu): 5, and of positively charged residues (Arg + Lys): 0
** Total number of negatively charged residues (Asp + Glu): 6, and of positively charged residues (Arg + Lys): 1

Both proteins lack glycine, and ORF7b1 shows a proline. The computed data were obtained from ProtParam (Expasy; https://web.expasy.org/protparam/).

Table S3 – Protein Sequence

Protein	Sequence (5 10 15 20 25 30 35 40)
ORF7b-2	MIELSLID FYLCFLAFLLFLVLIMLIIFWF SLELQDHNETCHA
ORF7b-1	MNELTLID FYLCFLAFLLFLVLIMLIIFWF SLEIQDLEEPCTKV

Note: The residues in red have a significant statistical tendency to form alpha-helices, green for coils, and blue for extended structures [69]. Residues 9 to 30, which are in larger characters, are those expected to form helices and be transmembrane. Therefore, both proteins' first 8-9 residues and the last 14-15 residues are involved in the terminal segments. Overall, only 46% of the residues in the entire molecule have a propensity for forming helices, but among the 20 residues shown as transmembrane helices, only 9 show sufficient helical propensity. The two proteins share the same sequence from positions 9 to 29. Visual analysis of the N-terminal sequences reveals the absence of any signal sequences (translocon sequences) [36]. Signal sequences are N-terminal extensions of nascent polypeptides (pre-proteins) in secretory and membrane proteins. They typically comprise about 15-30 amino acids and include a positively charged N-terminal region and a cleavage site for signal peptidase (Ala-X-Ala motif at the C-terminal end of the signal peptide). Neither protein exhibits the features [37] necessary for entry into the ER.

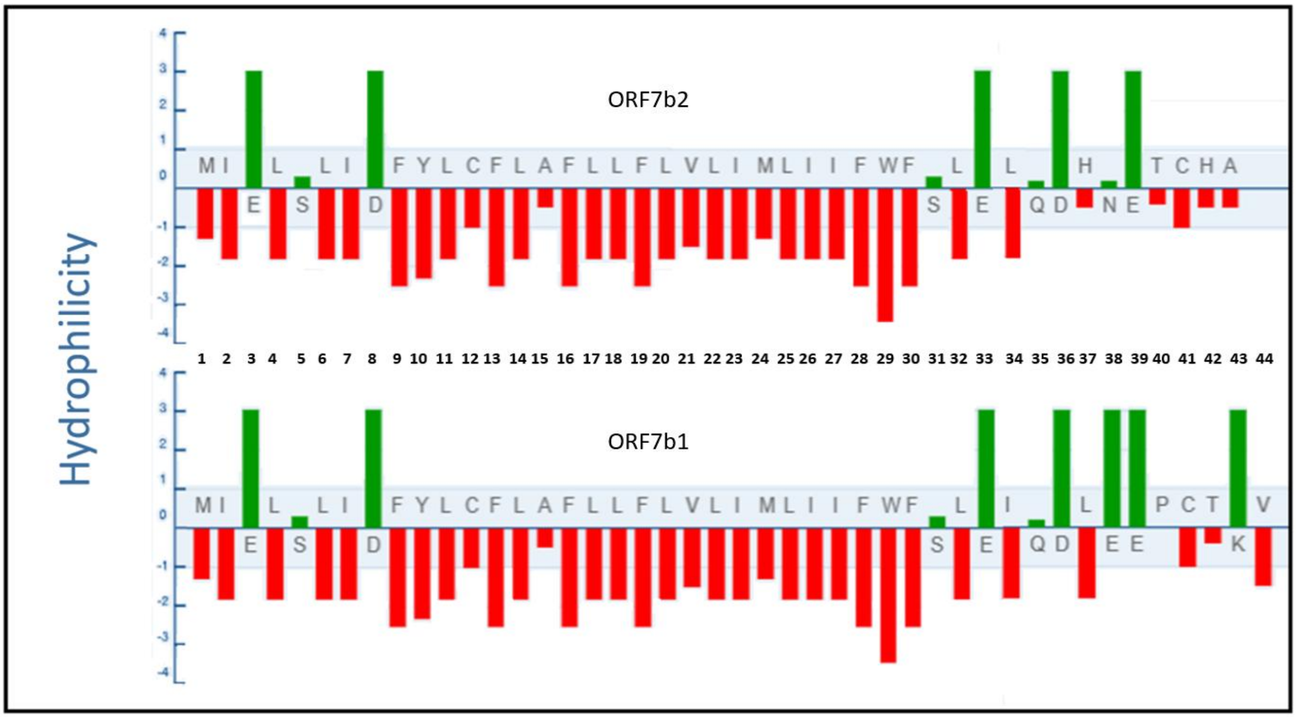

Figure S1 – Representation of the hydrophilicity of ORF7b2 and ORF7b1 along the sequence. The scale ranges from 4 to -4. Hydrophilic residues are shown in green, hydrophobic residues in red. The values are those of Kyte and Doolittle [X]. In both proteins, the terminal segments contain hydrophilic residues, with a higher concentration in the N-terminal segment. The central hydrophobic segment of both proteins comprises the same 22 residues (residues 9-30). The C-terminal segment of ORF7b1 is more hydrophilic and contains a proline at position 40 and a lysine at position 43, the only positive charge in the sequence.

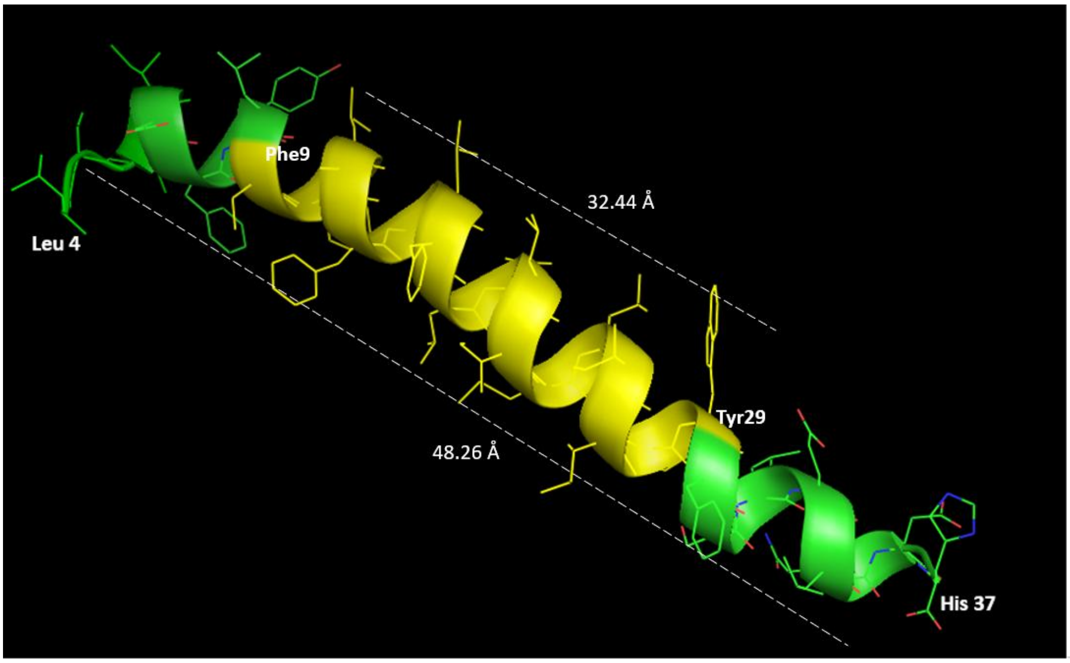

Figure S2 – **UCSF 3D-model**. One of the most reputable models is the 3D structure of ORF7b-2 from ModBase (University of California, San Francisco–UCSF). The model displays a single, long alpha helix. The prediction excludes terminal residues. Segment 9-29 (highlighted in yellow) represents the part of the helix that is likely transmembrane. The rigid visualization of the helix, without accounting for its conformational dynamics, electrostatic properties, or inter-residue distances, is probably the main reason various authors consider the protein to be transmembrane. Template PDB code: 4uvmA (template region: 478-511, with 29% sequence identity). Predicted residues: 4 to 37. Model available from PyMOL as a PNG.

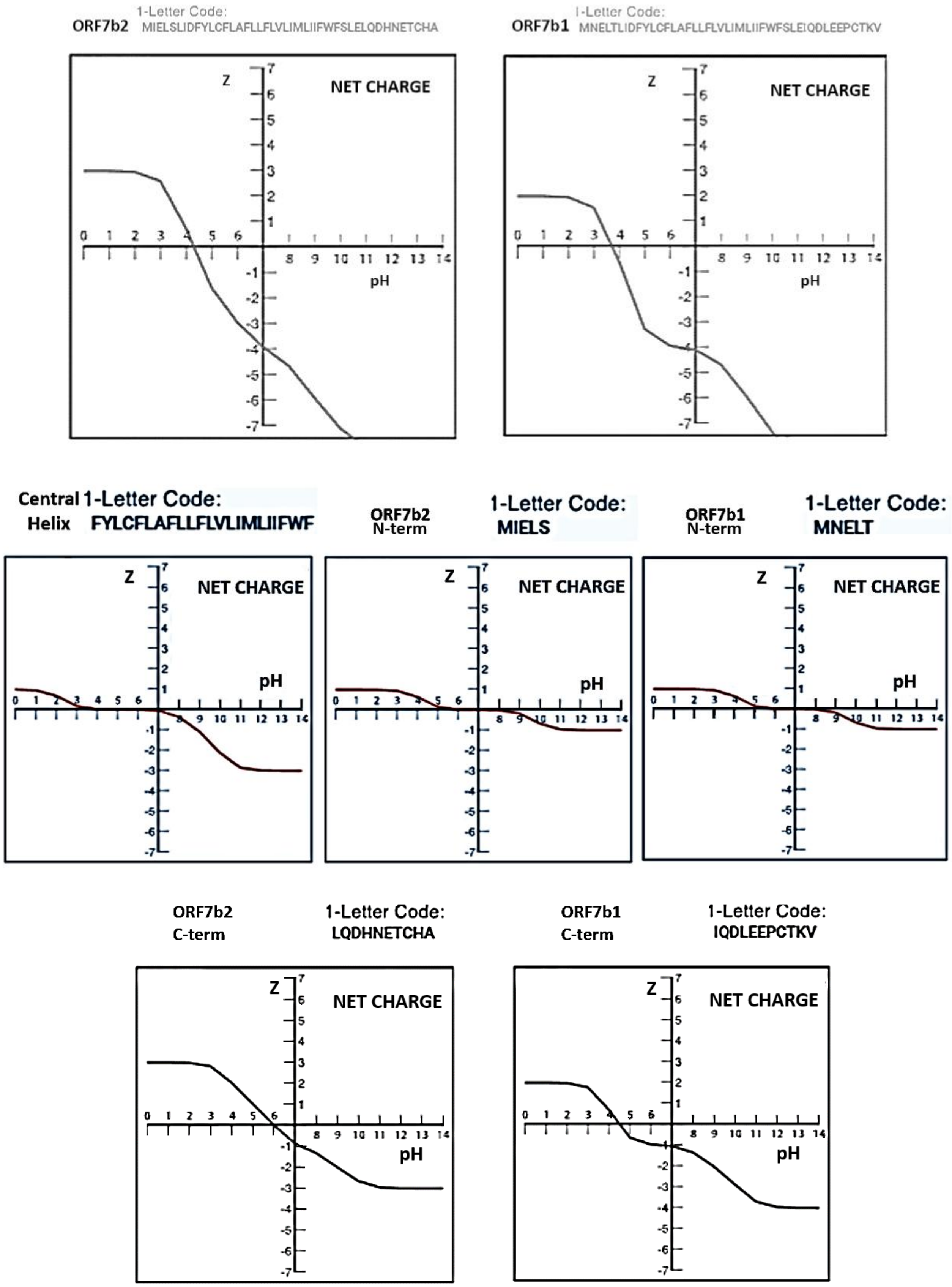

Figure S3 – pH dependence of specific segments of ORF7b1 and ORF7b2. The figures above show protein behavior and serve as a reference. The article text already described them. How the net charge depends on pH for the central segment, which is the same for both proteins (figure on the left, middle row), two N-terminal tails, and two C-terminal tails, is shown in the remaining figures.

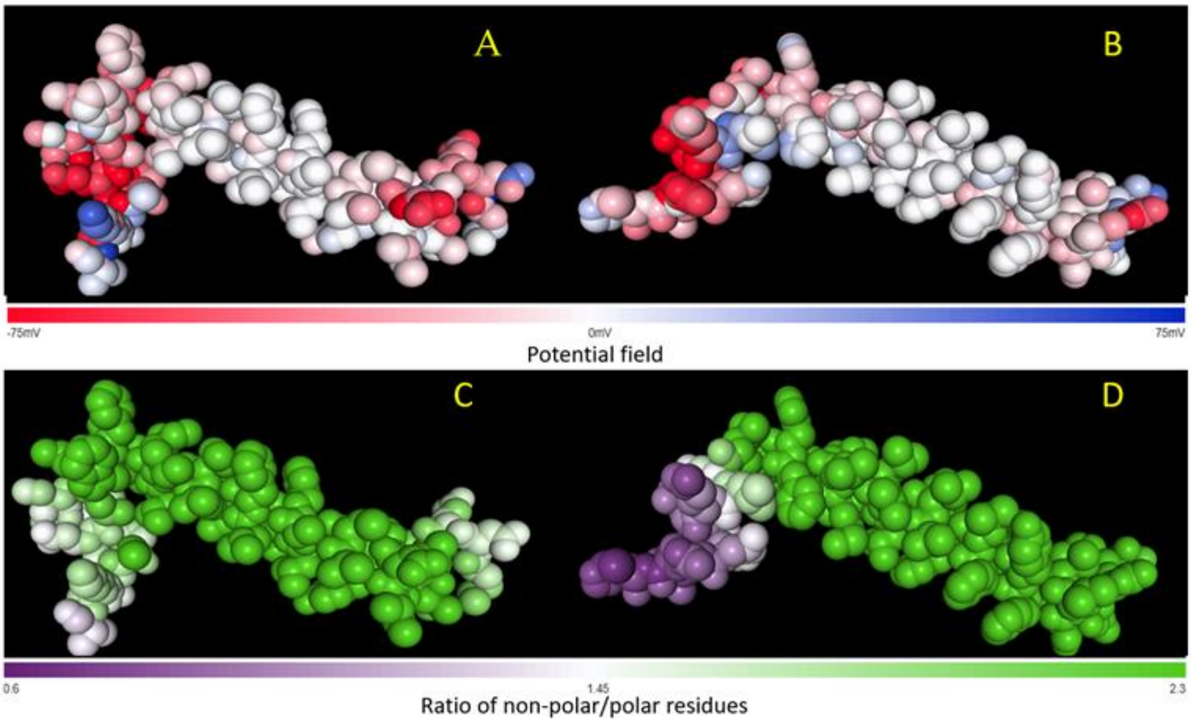

Figure S4 – ESPA and NPP distributions. Top, distribution of electrostatic surface potentials for the atoms of ORF7b1 (A) and ORF7b2 (B). At the **bottom**, the figure shows the NPP ratio for each atom of ORF7b1 (C) and ORF7b2 (D). The potential color code accompanies both distributions in the plot. The representation is space-filled. The NPP ratio reveals marked differences in polarity between the two molecules compared with predictions from simpler charge-per-atom models. The University of Manchester (UK) web server (https://protein-sol.manchester.ac.uk/) supports these evaluations by modeling 3D protein structure as a starting point. This system generates maps that show how pH and ionic strength influence a protein's folding stability. It also uses ionizable-group interactions and pKa calculations via the Debye-Hückel (DH) method, thereby directly linking pH-dependent stability to electrical charge [51-53]. The server also enables the reconstruction of three-dimensional structural models and assigns a specific structural category to each atom. These categories are defined based on the solvent-accessible surface area (SASA) calculated for each atom, which indicates the extent to which individual atoms are exposed to the surrounding solvent [54]. In addition, the server calculates the nonpolar-to-polar (NPP) ratio and the electrostatic surface potential (ESPA) of atoms, namely the electrical charge associated with each atom at the molecular surface [49,52]. The combined use of these two parameters is informative, as it enables a more accurate comparison of the actual electrostatic surface properties of the two molecules.

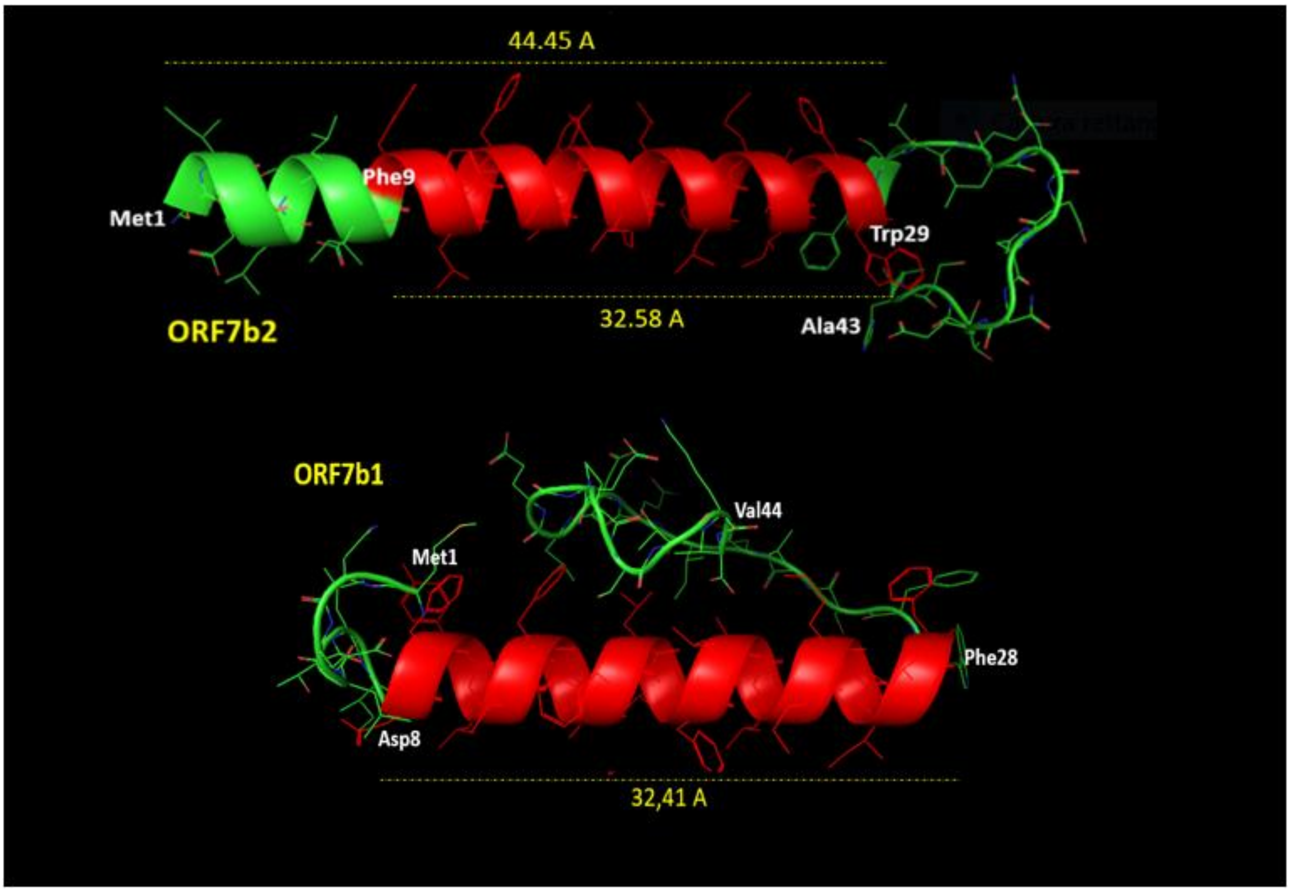

Figure S5 – PEP-FOLD3 models of ORF7b-2 and ORF7b-1. The figure shows the dimensions in Å of the two proteins. Molecular dynamics simulations show that segments 1-9 are highly mobile around the hinge residue Phe9. Models from PyMOL are in PNG format.

Table S4 – a) ORF7b-2 - PHYRE2 - Template analysis

Detailed template information

#	Template	Alignment Coverage	3D Model	Confidence	% i.d.	Template Information
1	c2zxeB_	Alignment	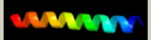	88.0	21	**PDB header:**hydrolase/transport protein<br>**Chain:** B: **PDB Molecule:**na+,k+-atpase beta subunit;<br>**PDBTitle:** crystal structure of the sodium - potassium pump in the e2.2k+.pi2 state<br>**PDB Entry:** PDBe RCSB PDBj
2	c7wyvB_	Alignment	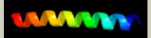	85.8	21	**PDB header:**membrane protein<br>**Chain:** B: **PDB Molecule:**na+,k+-atpase beta subunit;<br>**PDBTitle:** cryo-em structure of na+,k+-atpase in the e2p state formed by atp in2 the presence of 40 mm mg2+<br>**PDB Entry:** PDBe RCSB PDBj
3	c7wytB_	Alignment	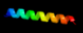	85.0	14	**PDB header:**membrane protein<br>**Chain:** B: **PDB Molecule:**sodium/potassium-transporting atpase subunit beta-1;<br>**PDBTitle:** crystal structures of na+,k+-atpase in complex with ouabain<br>**PDB Entry:** PDBe RCSB PDBj
4	c7e21B_	Alignment	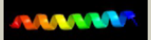	81.1	15	**PDB header:**membrane protein<br>**Chain:** B: **PDB Molecule:**sodium/potassium-transporting atpase subunit beta-1;<br>**PDBTitle:** cryo em structure of a na+-bound na+,k+-atpase in the e1 state with2 atp-gamma-s<br>**PDB Entry:** PDBe RCSB PDBj

Domain analysis

Rank	Aligned region
1	c2zxeB_
2	c7wyvB_
3	c7wytB_
4	c7e21B_
5	c3kdpD_
6	c2yn9B_
7	c2xzbB_
8	c3b8eB_
9	c7vu5B_
10	c7vu5A_
11	c2kogA_
12	c6lumO_
13	c6lumE_
14	c6lumI_
15	c2n1pA_
16	c7k3gE_
17	c7k3gA_
18	c7k3gC_
19	c7k3gB_
20	c7k3gD_

Table S4 - b) ORF7b-1 - PHYRE2 - Template analysis

Detailed template information

#	Template	Alignment Coverage	3D Model	Confidence	% i.d.	Template Information
1	c2zxeB_	Alignment		86.4	22	**PDB header:**hydrolase/transport protein **Chain:** B: **PDB Molecule:**na+,k+-atpase beta subunit; **PDBTitle:** crystal structure of the sodium - potassium pump in the e2.2k+.pi2 state **PDB Entry:** PDBe RCSB PDBj
2	c7wyvB_	Alignment		83.9	22	**PDB header:**membrane protein **Chain:** B: **PDB Molecule:**na+,k+-atpase beta subunit; **PDBTitle:** cryo-em structure of na+,k+-atpase in the e2p state formed by atp in2 the presence of 40 mm mg2+ **PDB Entry:** PDBe RCSB PDBj
3	c7wytB_	Alignment		83.2	15	**PDB header:**membrane protein **Chain:** B: **PDB Molecule:**sodium/potassium-transporting atpase subunit beta-1; **PDBTitle:** crystal structures of na+,k+-atpase in complex with ouabain **PDB Entry:** PDBe RCSB PDBj
4	c7e21B_	Alignment		78.5	15	**PDB header:**membrane protein **Chain:** B: **PDB Molecule:**sodium/potassium-transporting atpase subunit beta-1; **PDBTitle:** cryo em structure of a na+-bound na+,k+-atpase in the e1 state with2 atp-gamma-s **PDB Entry:** PDBe RCSB PDBj

Domain analysis

Rank	Aligned region
1	c2zxeB_
2	c7wyvB_
3	c7wytB_
4	c7e21B_
5	c3kdpD_
6	c2yn9B_
7	c2xzbB_
8	c3b8eB_
9	c7vu5B_
10	c7vu5A_
11	c2kogA_
12	c7k3gC_
13	c7k3gA_
14	c7k3gB_
15	c7k3gE_
16	c7k3gD_
17	c6lumO_
18	c6lumE_
19	c6lumI_
20	c2n1pA_

Note: The domain analysis displays the locations of matches along the sequence, with color-coding showing confidence levels. An alignment that considers the number of aligned residues and the quality of the match ranks the results. This ranking is based on the similarity of residue probability distributions at each position, secondary structure similarity, and the presence or absence of insertions and deletions. Each row shows details about the template used for the model and includes a small graphic illustrating where the match occurs along your sequence, color-coded by confidence.

Table S5 – a) ORF7b-2 (PHYRE2 model) – Ramachandran statistics

Protein Geometry	Poor rotamers	0	0.00%	Goal: <0.3%
	Favored rotamers	41	100.00%	Goal: >98%
	Ramachandran outliers	0	0.00%	Goal: <0.05%
	Ramachandran favored	37	90.24%	Goal: >98%
	Rama distribution Z-score	1.40 ± 1.09		Goal: abs(Z score) < 2
	Cβ deviations >0.25Å	0	0.00%	Goal: 0
	Bad bonds:	4 / 375	1.07%	Goal: 0%
	Bad angles:	2 / 509	0.39%	Goal: <0.1%

Table S5 - b) ORF7b-2 (PEP-FOLD3 model) – Ramachandran statistics

Protein Geometry	Poor rotamers	0	0.00%	Goal: <0.3%
	Favored rotamers	35	97.22%	Goal: >98%
	Ramachandran outliers	1	2.44%	Goal: <0.05%
	Ramachandran favored	36	87.80%	Goal: >98%
	Rama distribution Z-score	0.92 ± 1.15		Goal: abs(Z score) < 2
	Cβ deviations >0.25Å	0	0.00%	Goal: 0
	Bad bonds:	5 / 373	1.34%	Goal: 0%
	Bad angles:	3 / 506	0.59%	Goal: <0.1%
Peptide Omegas	Cis Prolines:	0 / 0	0.00%	Expected: ≤1 per chain, or ≤5%
Low-resolution Criteria	CaBLAM outliers	1	2.6%	Goal: <1.0%
	CA Geometry outliers	0	0.00%	Goal: <0.5%
Additional validations	Chiral volume outliers	0/61		

Table S5 - c) ORF7b-1 (PHYRE2 model) – Ramachandran statistics

Protein Geometry	Poor rotamers	0	0.00%	Goal: <0.3%
	Favored rotamers	43	100.00%	Goal: >98%
	Ramachandran outliers	1	2.38%	Goal: <0.05%
	Ramachandran favored	38	90.48%	Goal: >98%
	Rama distribution Z-score	-6.78 ± 0.80		Goal: abs(Z score) < 2
	Cβ deviations >0.25Å	0	0.00%	Goal: 0
	Bad bonds:	12 / 381	3.15%	Goal: 0%
	Bad angles:	21 / 517	4.06%	Goal: <0.1%
Peptide Omegas	Cis Prolines:	0 / 1	0.00%	Expected: ≤1 per chain, or ≤5%
Low-resolution Criteria	CaBLAM outliers	0	0.0%	Goal: <1.0%
	CA Geometry outliers	0	0.00%	Goal: <0.5%
Additional validations	Chiral volume outliers	0/64		

Table S5 - d) ORF7b-1 (PEP-FOLD3 model) – Ramachandran statistics

Protein Geometry	Poor rotamers	2	5.26%	Goal: <0.3%
	Favored rotamers	35	92.11%	Goal: >98%
	Ramachandran outliers	3	7.14%	Goal: <0.05%
	Ramachandran favored	34	80.95%	Goal: >98%
	Rama distribution Z-score	-0.52 ± 1.18		Goal: abs(Z score) < 2
	Cβ deviations >0.25Å	0	0.00%	Goal: 0
	Bad bonds:	1 / 380	0.26%	Goal: 0%
	Bad angles:	0 / 516	0.00%	Goal: <0.1%
Peptide Omegas	Cis Prolines:	0 / 1	0.00%	Expected: ≤1 per chain, or ≤5%
Low-resolution Criteria	CaBLAM outliers	4	10.0%	Goal: <1.0%
	CA Geometry outliers	0	0.00%	Goal: <0.5%
Additional validations	Chiral volume outliers	0/64		

Note: The Tables S5 show the statistical analysis of the best PHYRE2 (a and b) and PEP-FOLD3 (c and d) models, based on the Ramachandran plot.

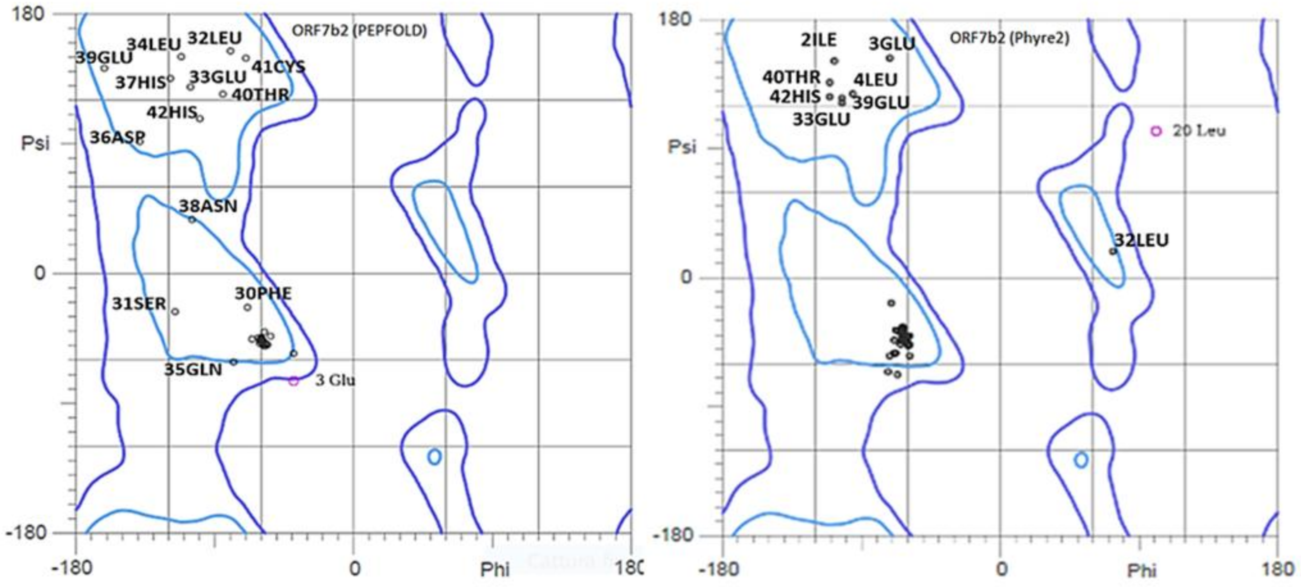

Figure S6 a– Ramachandran plots of the two 3D models of ORF7b2. The residues with abnormal angles in the “extended” zone are all located in the terminal sequences. Both modeling methods produced similar results. Correctly alpha-helical residues are mainly found in the alpha zone [Φ -60° and Ψ -50°]. 3 Glu (to the left) and 20 Leu (to the right) are outlier residues.

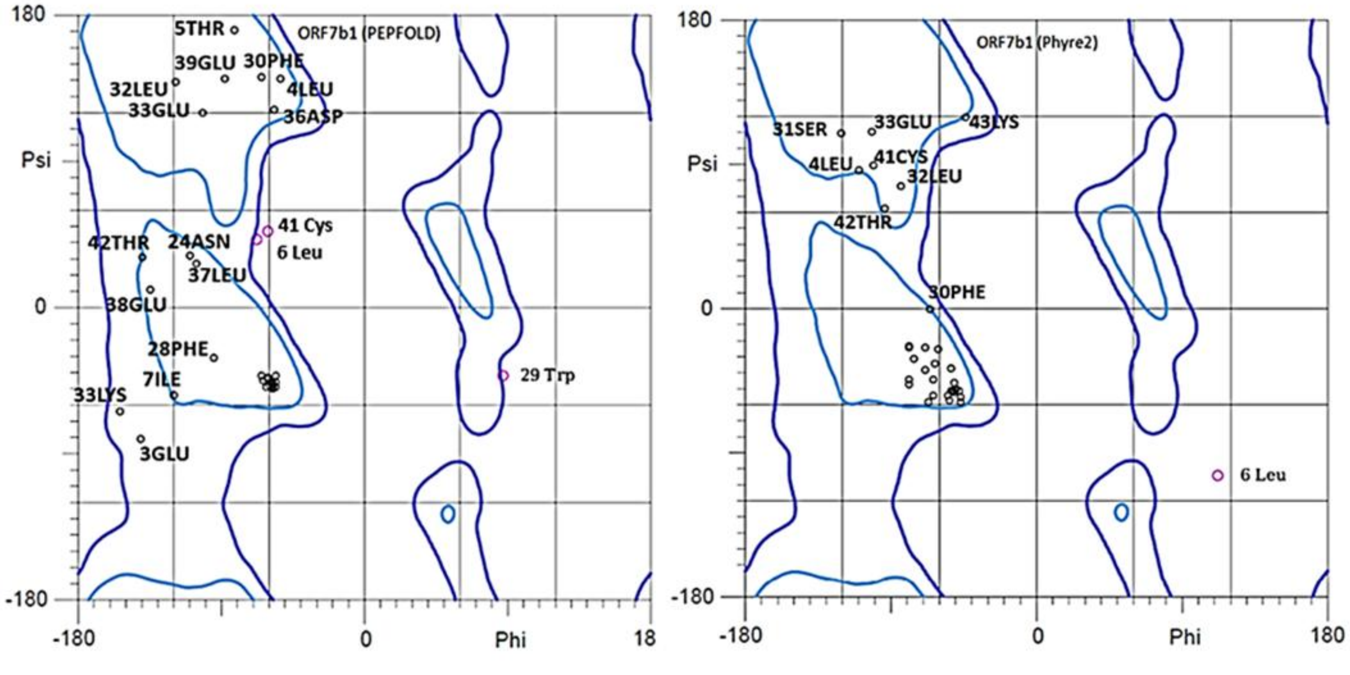

Figure S6 b – Ramachandran plots of the two 3D models of ORF7b1. Residues with unusual angles are widely dispersed, and many occur in terminal sequences. Residues in red are outliers (41 Cys, 6 Leu, and 29 Trp, to the left and 6 Leu to the right).

Table S6–Molecular contacts calculated by RING4 for the ORF7b2 and ORF7b1 models.

ORF7b2					ORF7b1				
H-bond			van der Waals		H-bond			van der Waals	
Source	Target	Seq	Source	Target	Source	Target	Seq	Source	Target
		3	3/GLU	6/LEU	5/THR	9/PHE	5	5/THR	9/PHE
5/SER	9/PHE	5					6	6/LEU	10/TYR
		6	6/LEU	9/PHE	7/ILE	11/LEU	7		
7/ILE	11/LEU	7	7/ILE	11/LEU			8	8/ASP	12/CYS
8/ASP	12/CYS	8			**9/PHE**	**12/CYS**	9	**9/PHE**	**13/PHE**
9/PHE	13/PHE	9			**9/PHE**	**13/PHE**	-		
10/TYR	14/LEU	10	10/TYR	13/PHE	10/TYR	14/LEU	10	10/TYR	13/PHE
			10/TYR	14/LEU				10/TYR	14/LEU
11/LEU	15/ALA	11	11/LEU	15/ALA	11/LEU	15/ALA	11	11/LEU	15/ALA
12/CYS	16/PHE	12			**12/CYS**	**16/PHE**	12		
**13/PHE**	**17/LEU**	13			**13/PHE**	**17/LEU**	13	13/PHE	16/PHE
14/LEU	17/LEU	14			14/LEU	18/LEU	14	14/LEU	17/LEU
14/LEU	18/LEU	-			15/ALA	18/LEU	15	15/ALA	18/LEU
15/ALA	18/LEU	15			15/ALA	19/PHE			
15/ALA	19/PHE	-					16	**16/PHE**	**19/PHE**
16/PHE	20/LEU	16			16/PHE	20/LEU			
**17/LEU**	**21/VAL**	17	**17/LEU**	**20/LEU**	**17/LEU**	**21/VAL**	17		
**18/LEU**	**22/LEU**	18	**18/LEU**	**21/VAL**	18/LEU	22/LEU	18		
		-			**19/PHE**	**23/ILE**	19		
19/PHE	23/ILE	19			20/LEU	24/MET	20	20/LEU	23/ILE
20/LEU	23/ILE	20	20/LEU	24/MET	**21/VAL**	**25/LEU**	21		
20/LEU	24/MET	-			22/LEU	26/ILE	22	22/LEU	25/LEU
**21/VAL**	**25/LEU**	21	**21/VAL**	**25/LEU**	**23/ILE**	**26/ILE**	23	**23/ILE**	**26/ILE**
**22/LEU**	**26/ILE**	22	**22/LEU**	**25/LEU**	23/ILE	27/ILE			
		-	**22/LEU**	**26/ILE**	24/MET	27/ILE	24	24/MET	27/ILE
**23/ILE**	**26/ILE**	23	**23/ILE**	**26/ILE**	24/MET	28/PHE		24/MET	28/PHE
23/ILE	27/ILE	-			25/LEU	28/PHE	25		
		-			25/LEU	29/TRP			
24/MET	28/PHE	24			26/ILE	29/TRP	26	26/ILE	29/TRP
25/LEU	28/PHE	25	25/LEU	22/LEU	26/ILE	30/PHE			
25/LEU	29/TRP	-	25/LEU	28/PHE			27	27/ILE	30/PHE
26/ILE	30PHE	26			28/PHE	31/SER	28		
27/ILE	30/PHE	27					29	29/TRP	37/LEU
27/ILE	31/SER	-			33/GLU	36/ASP	33	33/GLU	36/ASP
28/PHE	32/LEU	28	28/PHE	31/SER	33/GLU	37/LEU		33/GLU	37/LEU
33/GLU	37/HIS	33					34	34/ILE	38/GLU
34/LEU	38/ASN	34	34/LEU	38/ASN	36/ASP	39/GLU	36		
35/GLN	38/ASN	35	35/GLN	38/ASN	37/LEU	41/CYS	37	37/LEU	40/PRO
35/GLN	39/GLU	-							

Note: The table reports pairs of residues that interact via hydrogen bonds and van der Waals contacts for ORF7b1 and ORF7b2. According to Table 5, residues highlighted in red show a high degree (Hub) and high centrality.

Table S7 Calculated topological values for the RIN of ORF7b2 and ORFb1 models

ORF7b2			ORF7b1		
**Betweenness centrality**	**Degree**	**Residue**	**Betweenness centrality**	**Degree**	**Residue**
276.3333	4.0	22/LEU	142.3337	3.0	12/CYS
261.0	5.0	26/ILE	140.3377	3.0	16/PHE
194.0	4.0	23/ILE	126.3338	8.0	9/PHE
187.6666	3.0	17/LEU	117.0	5.0	23/ILE
155.9999	4.0	21/VAL	107.0	3.0	19/PHE
148.1666	5.0	25/LEU	104.0001	4.0	17/LEU
143.1666	4.0	20/LEU	103.0	5.0	13/PHE
142.4999	3.0	18/LEU	99.66660	3.0	25/LEU
126.0	2.0	19/PHE	92.0	4.0	26/ILE
92.1666	4.0	13/PHE	84.66070	2.0	21/VAL
88.0	3.0	15/ALA	65.66667	3.0	20/LEU
88.0	2.0	16/PHE	64.0	2.0	22/LEU
56.0	3.0	28/PHE	49.66666	5.0	14/LEU
51.0	3.0	27/ILE	42.0	3.0	15/ALA
48.3333	3.0	14/LEU	31.33333	3.0	24/MET
46.0	4.0	11/LEU	25.66644	3.0	28/PHE
46.0	3.0	9/PHE	19.33332	3.0	10/TYR
46.0	2.0	12/CYS	6.0	4.0	37/LEU
43.1666	3.0	24/MET	4.0	2.0	36/ASP
34.5767	3.0	10/TYR	4.0	2.0	39/GLU
34.0	2.0	30/PHE	0.0	1.0	29/TRP
0.0	2.0	7/ILE	0.0	2.0	40/PRO
0.0	1.0	3/GLU	0.0	1.0	41/CYS
0.0	1.0	5/SER	0.0	1.0	33/GLU
0.0	1.0	6/LEU	0.0	1.0	42/THR
0.0	1.0	8/ASP	0.0	1.0	8/ASP
0.0	1.0	31/SER	0.0	1.0	27/ILE
0.0	1.0	33/GLU	0.0	4.0	5/THR
0.0	1.0	34/LEU	0.0	1.0	30/PHE
0.0	1.0	35/GLN	0.0	2.0	11/LEU
0.0	1.0	37/HIS	0.0	1.0	18/LEU
0.0	1.0	38/ASN	0.0	0.0	1/MET
0.0	1.0	39/GLU	0.0	0.0	2/ASN
0.0	0.0	1/MET	0.0	0.0	3/GLU
0.0	0.0	2/ILE	0.0	0.0	31/SER
0.0	0.0	4/LEU	0.0	0.0	32/LEU
0.0	0.0	29/TRP	0.0	0.0	34/ILE
0.0	0.0	32/LEU	0.0	0.0	35/GLN
0.0	0.0	36/ASP	0.0	0.0	38/GLU
0.0	0.0	40/THR	0.0	0.0	4/LEU
0.0	0.0	41/CYS	0.0	0.0	43/LYS
0.0	0.0	42/HIS	0.0	0.0	44/VAL
0.0	0.0	43/ALA	0.0	0.0	6/LEU
			0.0	0.0	7/ILE

Note: Betweenness centrality measures how much a vertex lies on paths between other vertices. Vertices with high betweenness can have a significant influence within a network by managing information flow between others. A common approach is to select approximately 20% of nodes with the highest betweenness centrality values [75]. We chose the top 9 (21%) and 10 (22.7%). We show the selected nodes in red.

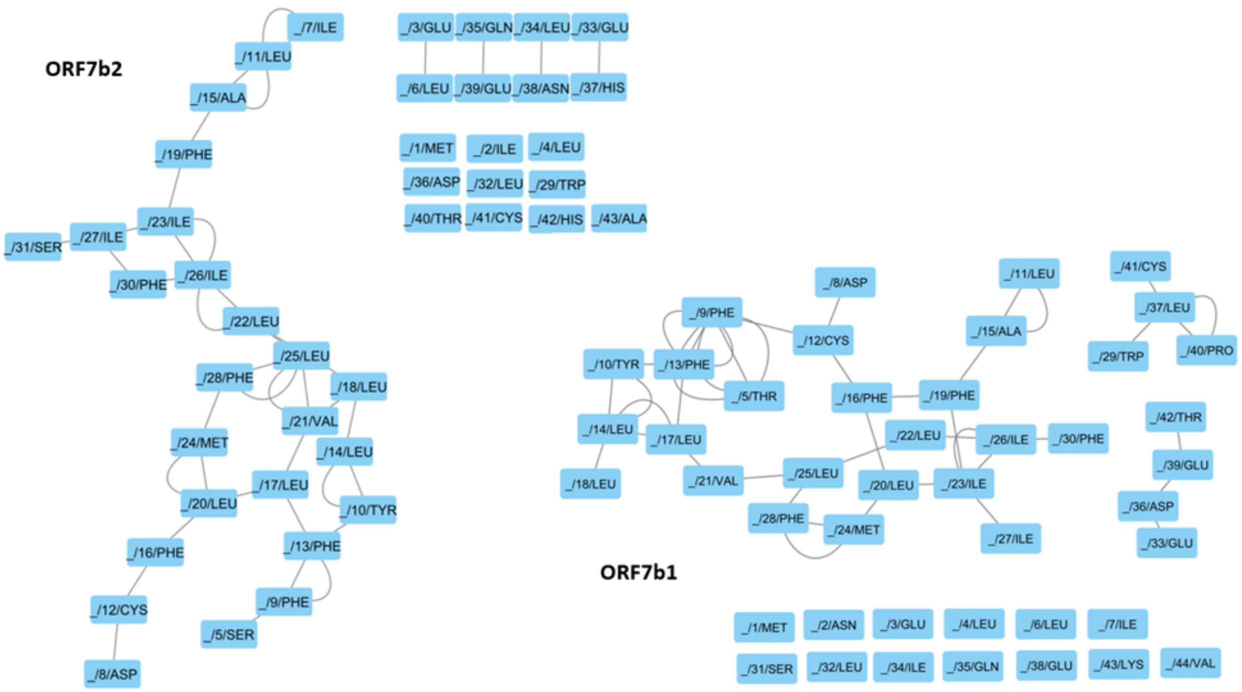

Figure S7 – Cytoscape representation of the two networks. The figure also displays all the unconnected residues of both proteins. A close view of these residues reveals that they are at the terminal ends of the two molecules. This finding is consistent with the 3D models.

Details of ORF7b-2 Dynamics

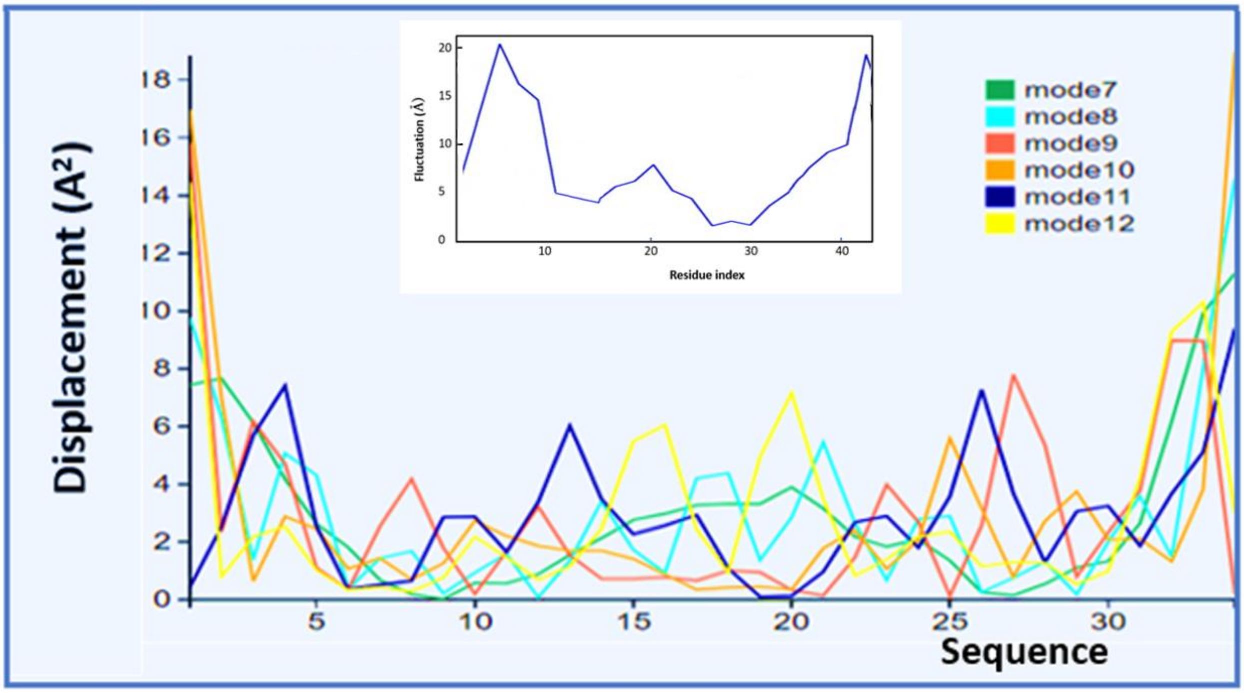

Figure S8 - Normal mode analysis (NMA) of ORF7b-2 - Mean residue index correlation from the analyzed modes. Central positions in the profile exhibit less model flexibility, whereas the outer segments are very flexible and unstable. The inset shows the average fluctuations calculated by elNémo from structures in Figure 12. The curve's trend shows a vibrational displacement of about 10 Å in the central segment.

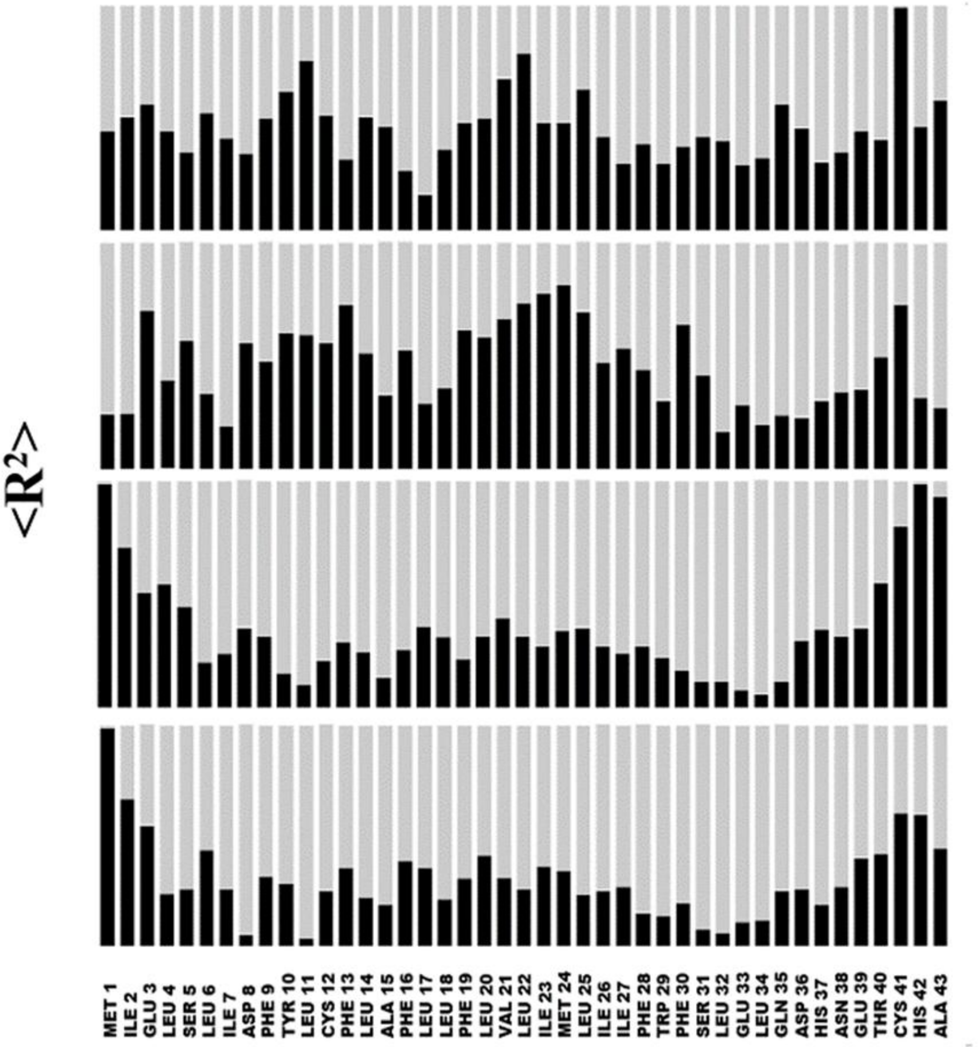

Figure S9 – Normal mode analysis (NMA) of ORF7b-2. The figure compares the displacement of a single residue for four selected modes: 1, 2, 8, and 7 (from top to bottom). The comparison between these four modes provides a dynamic view of residues, showing that even the central helical residues undergo conformational changes because of bending and other movements, twisting.

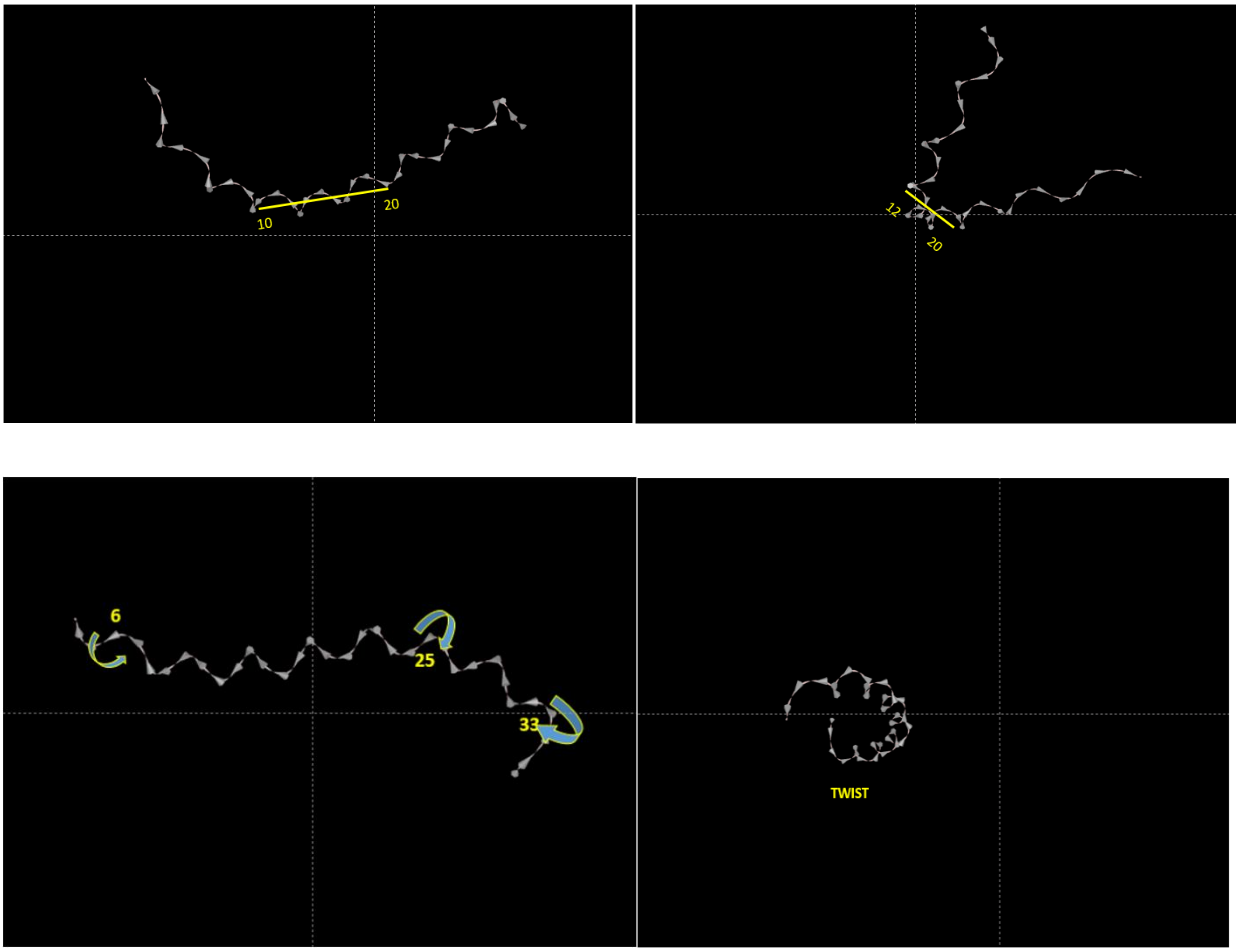

Figure S10 - Normal mode analysis (NMA) of ORF7b-2 - Some snapshots showing the conformational movements of ORF7b-2 calculated by dynamics (bending and twisting). The hinge residues are yellow.

Table S8

Dipole moment for ORF7b-2

	No. of Chains=1		Prolate							
	**No.Atoms**	**No.Res.**	**R_M**	**Pos.Res.**	**Neg.Res.**	**Charge**	**Dipole**	**Quadrapole**	**Crg./Nat.**	**Dip./Nat.**
**Value**	365.	43.	109.01	0.	5.	-5.	488.	1066.	-0.0137	1.3370
**No.Dev.Units**	-1.19	-1.22	-1.01	-1.47	-1.16	-0.23	-0.13	-0.42	-2.16	3.42

Dipole vector (in atomic units): -84.37 -16.03 54.30
Mass Moments vector: 230.47 106.03 206.34
Open a larger Jmol window.

The table shows the structural and physical parameters involved in the dipole calculation vector.

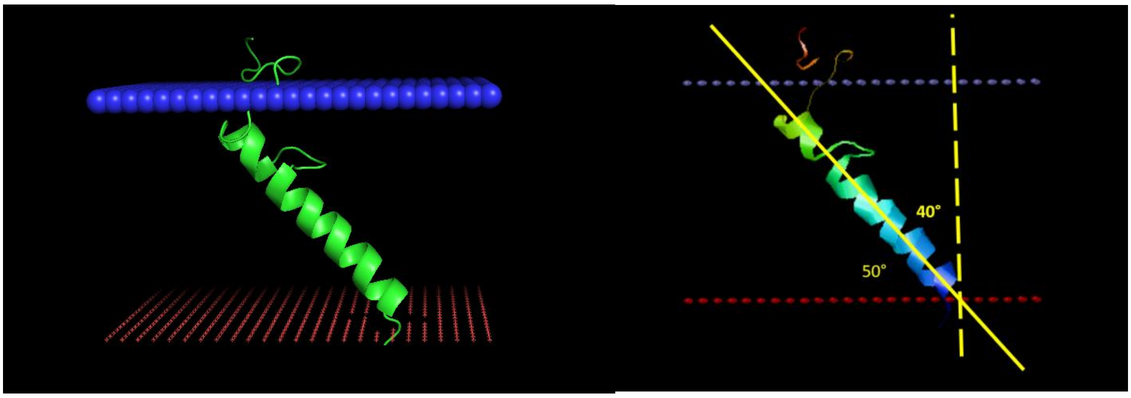

Figure S11 - Insertion of a single molecule of ORF7b-2 into a membrane. The figure illustrates another attempt to visualize the insertion of a single molecule of ORF7b-2 into a membrane using Memembed, an algorithm from the PSIPRED web server (see Methods). A tilt angle of 40° relative to the axis perpendicular to the membrane surface (right side) is displayed by the protein. The terminal segments extend outward from both the luminal and cytoplasmic sides, and the unfolding of some residues within the membrane shows structural stress (left side). Both the dipole moment, which depends on the tilt angle, and the solvent exposure of the terminal regions influence the overall dipole of the helix. This may explain the low value observed for ORF7b-2.

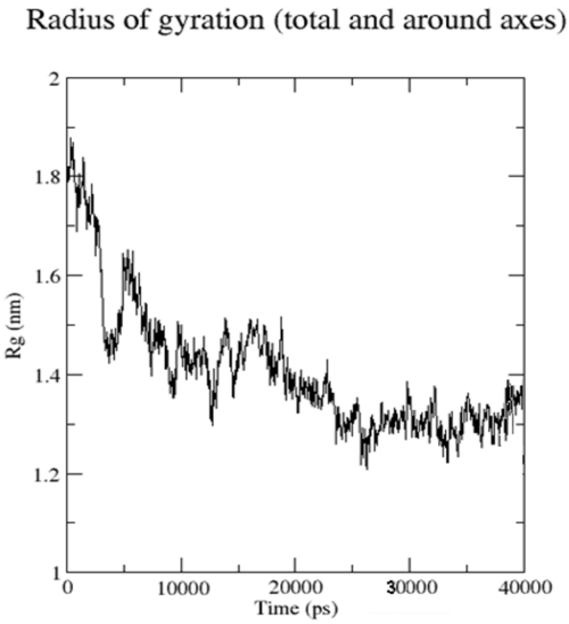

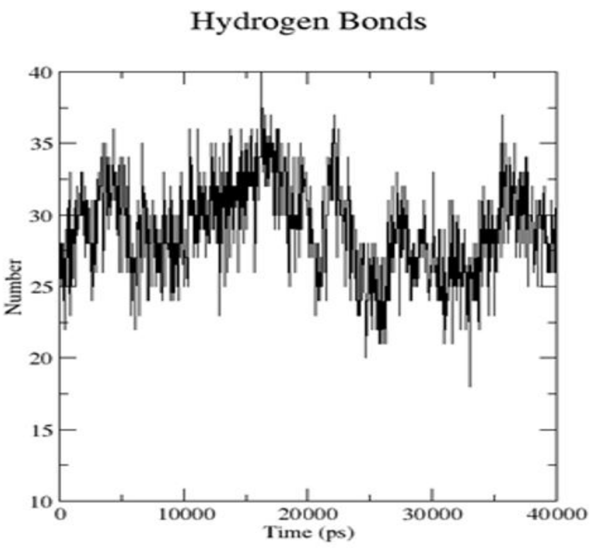

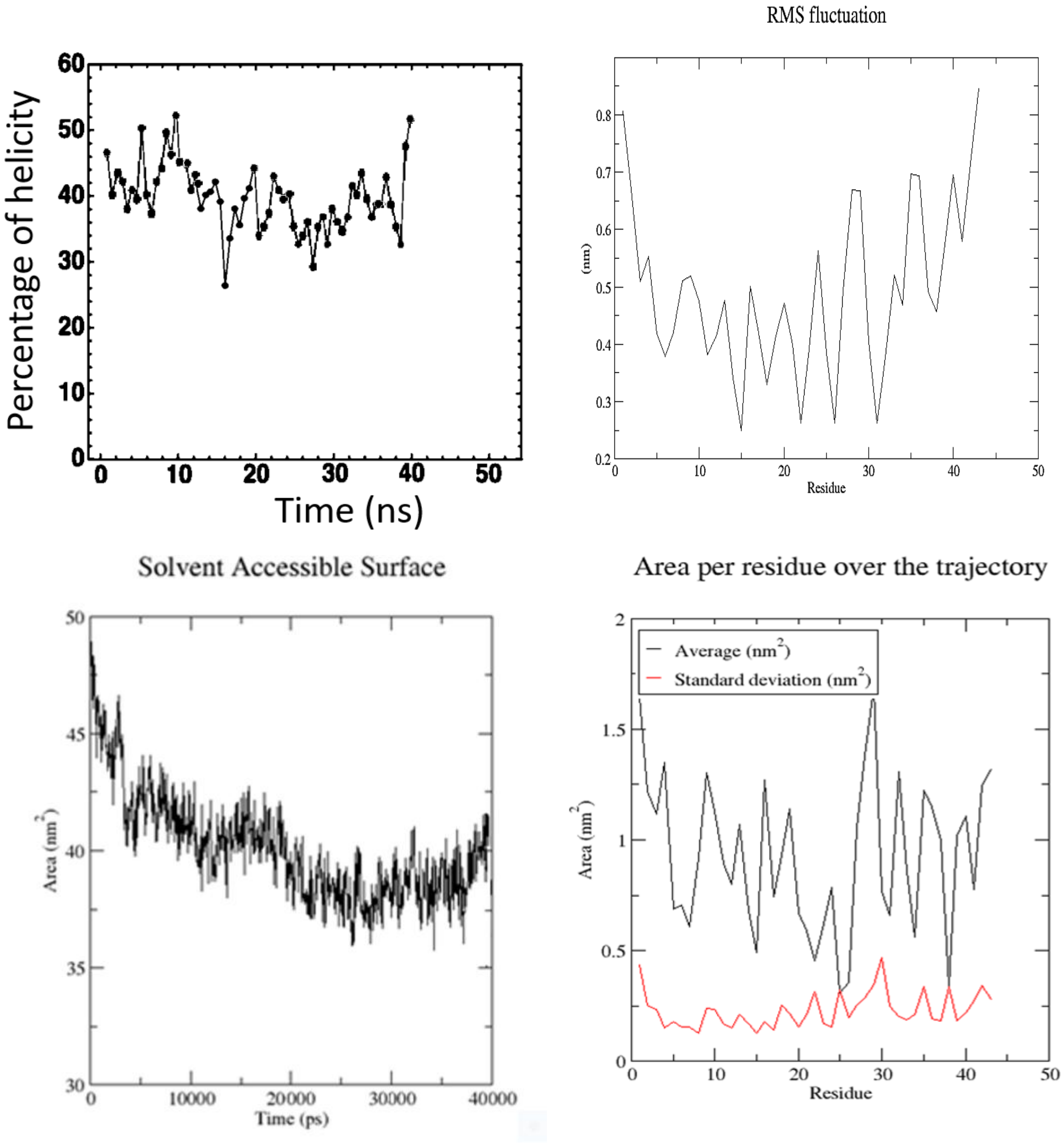

Figure S12 Parameters observed during the simulations. The figure shows the set of parameters (Rg, H bonds, helicity, RMS, ASA, and Area) that characterize the trend of the molecular dynamics simulation of ORF7b-2 in water for 40 ns. We repeated each measurement three times with very similar results.

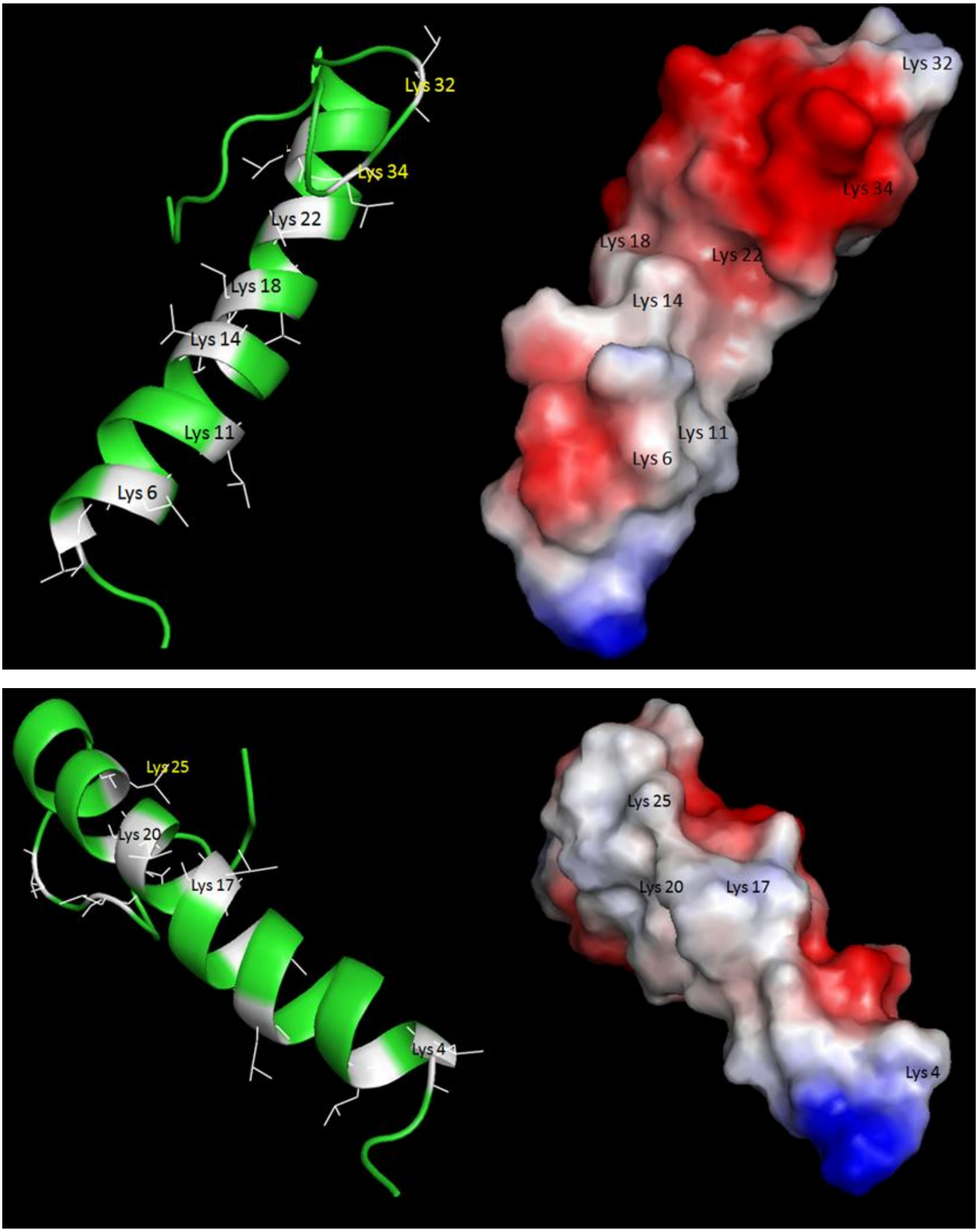

Figure S13 – Surface electrostatics - The two figures show the two sides of the structural model obtained at 40 ns through molecular dynamics in water. Different electrostatic environments on the molecule's opposite sides contain distributed lysines. Most are on the side with a large negative surface. Therefore, a Lys-zip with a linear sequence on a single apolar surface is physically absent.

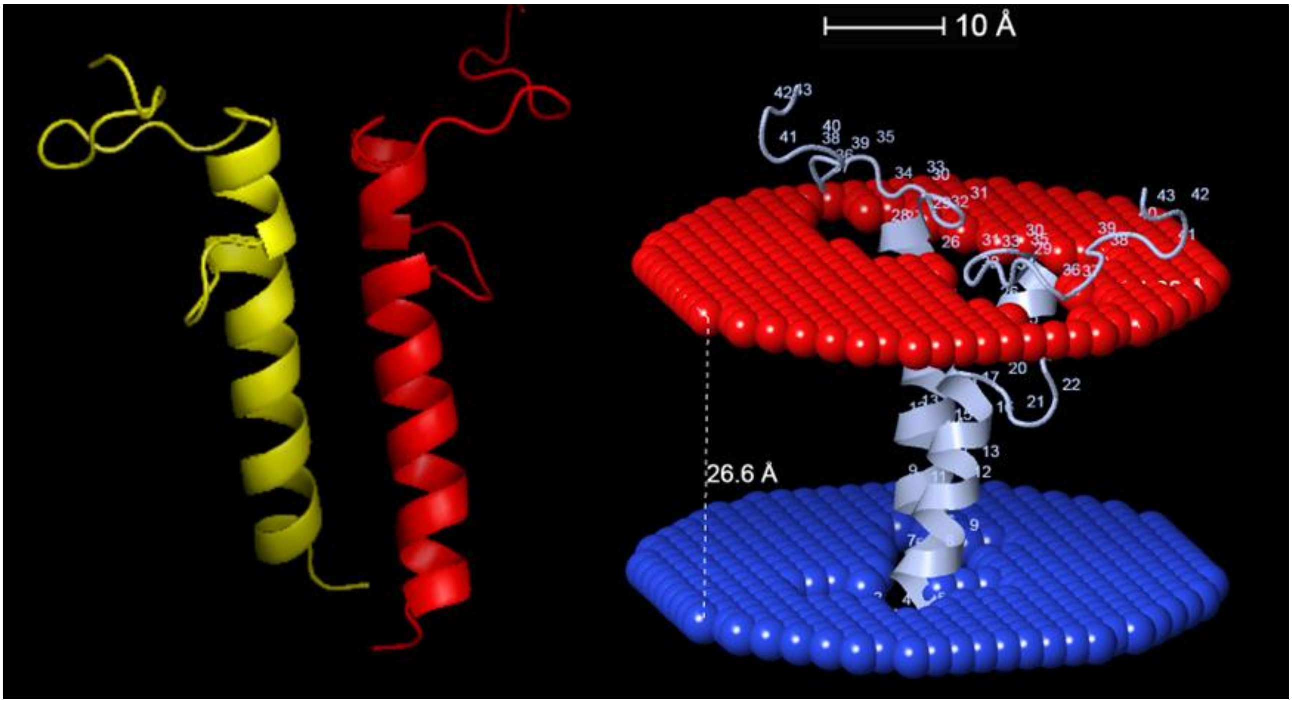

Figure S14 – Dimer models in the membrane. The figure shows, on the left, the model generated from HDOCK and, on the right, the model simulating the pre-orientation in the Golgi membrane obtained through the OPM database. In both models, a helix distortion is clear.

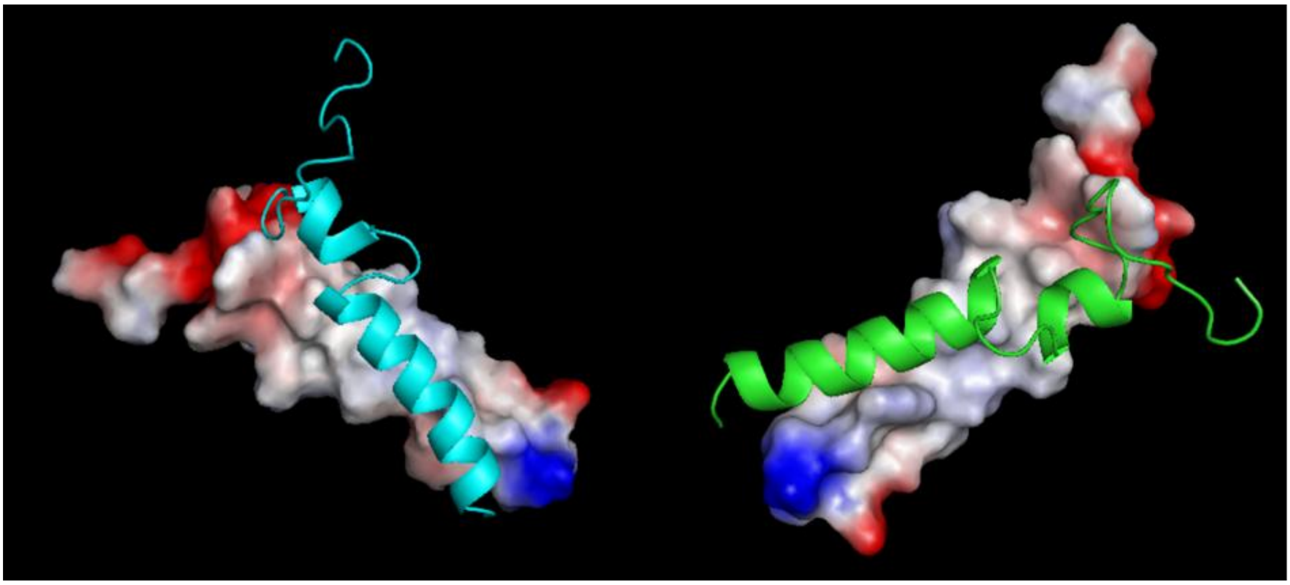

Figure S15 - Water-interaction interface. The figure depicts the parallel water-interaction interface between two ORF7b-2 molecules, as generated by HDOCK. The model shows that charged external surfaces protect two non-polar patches that mediate the main interaction. This structural configuration shows the internal dimeric apolar surface, with a broad distribution of external negative charge, which accounts for the dimer formation.

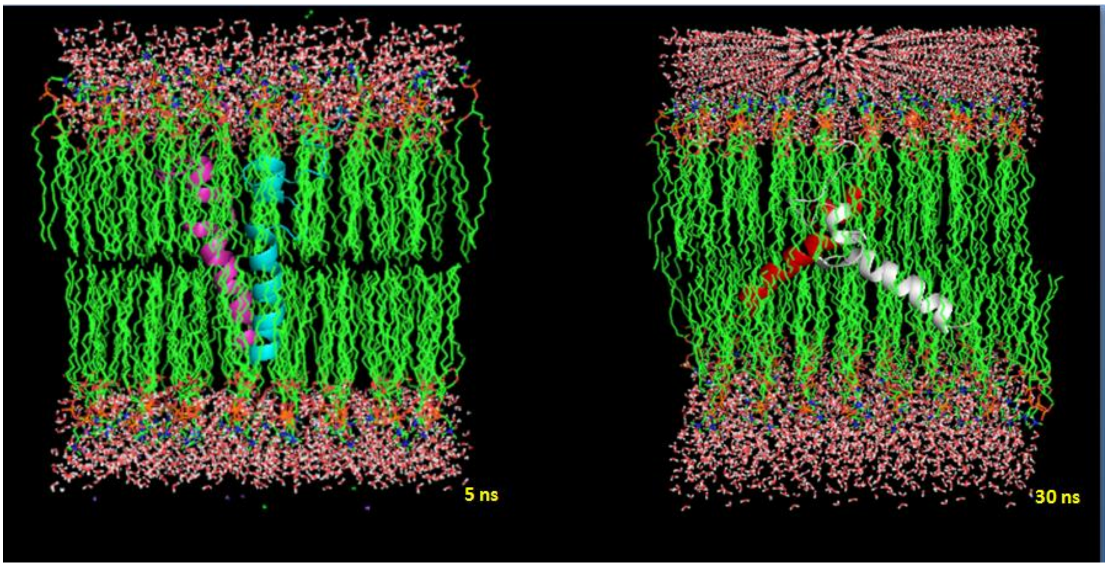
5 ns
30 ns

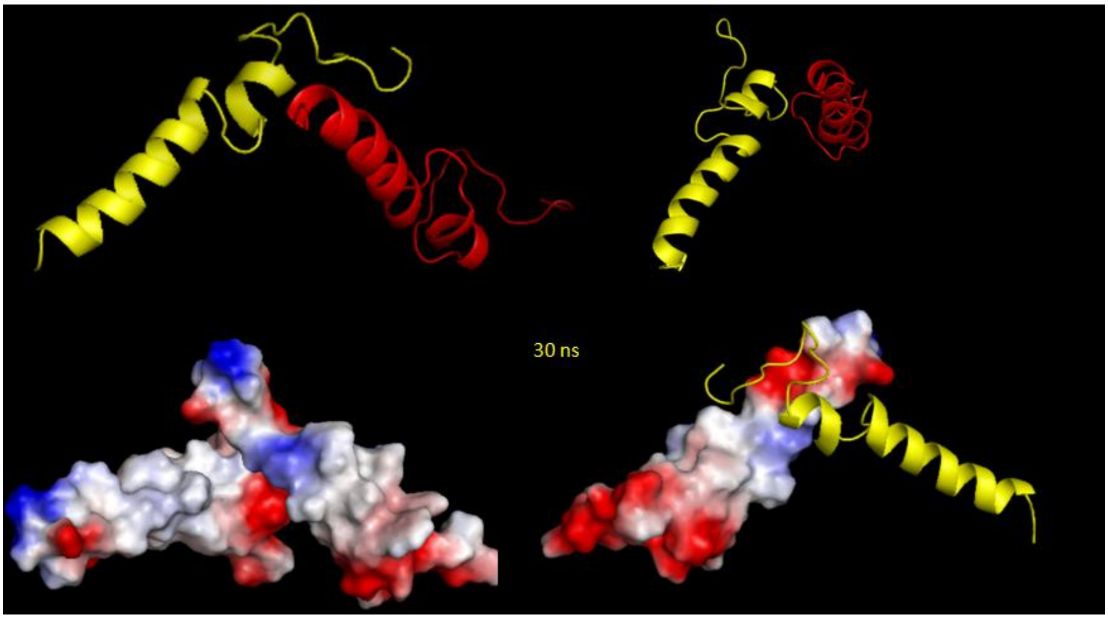
30 ns

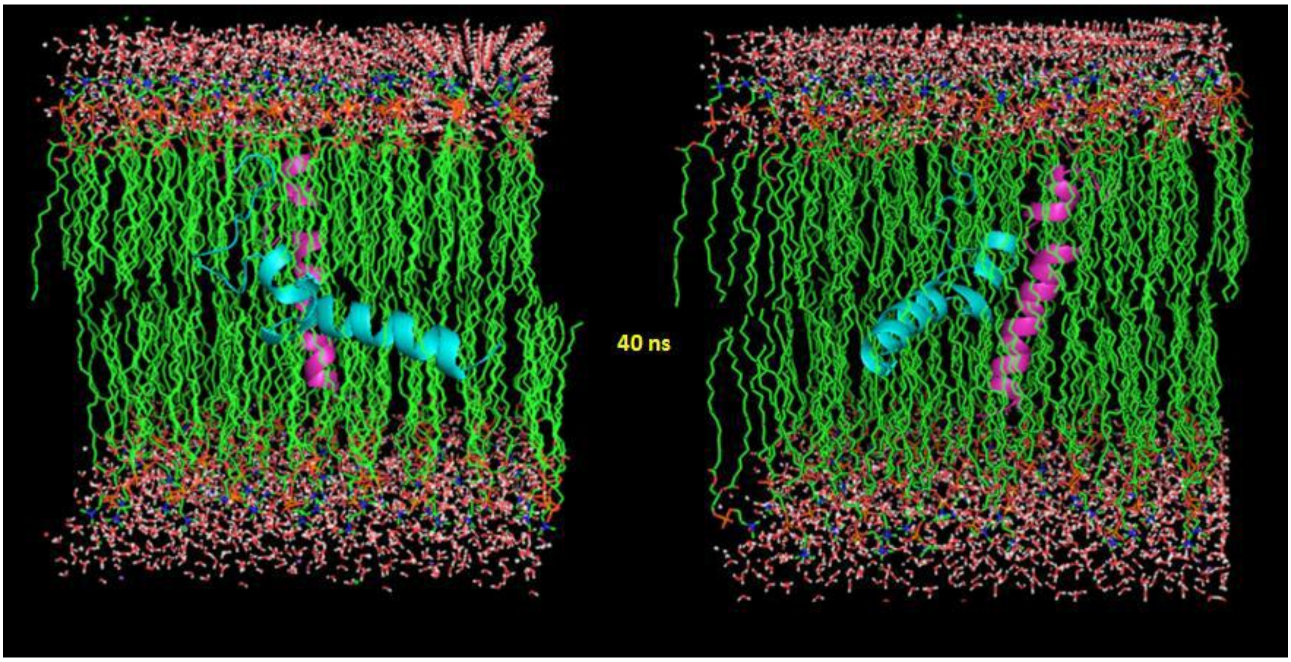
40 ns

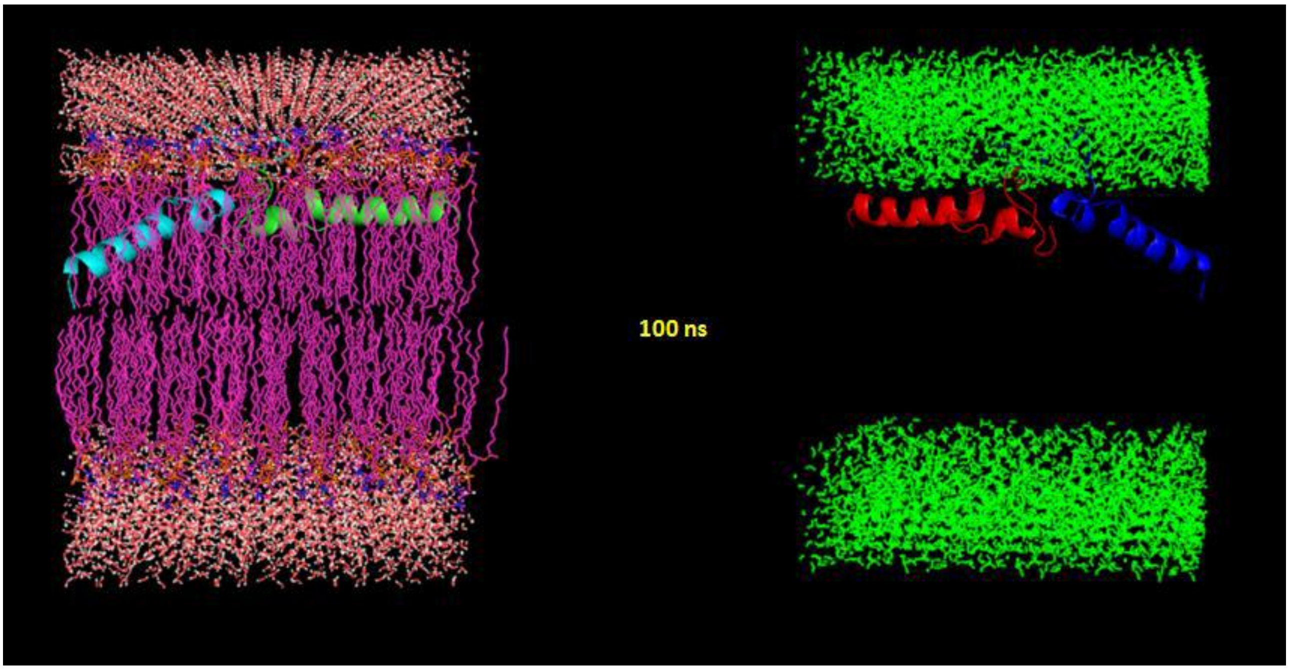

Figure S16 - Key steps of molecular dynamics in POPC. The figure depicts key steps in the dimer's evolution during a molecular dynamics simulation up to 100 ns. The different colors, or the absence of color, in the lipid sheets at 100 ns, are used to better illustrate the structural organization of the two ORF7b-2 molecules. At 70 ns, the structure was already organized and remained stable until 100 ns. ORF7b-2 rarely maintains a dimeric structure within the membrane. It gradually loses its structure and helical organization, orienting polar surfaces toward the lipid layer containing the polar heads. The details at 30 ns are interesting because they show how the distribution of electrostatic surfaces dynamically induces a head-to-tail (antiparallel) interaction between the two proteins, likely due to the negative charge of the C-terminus interacting with the positive charge of the N-terminus. This organization shows that the polar head layer attracts the two proteins, which move through rotational and translational motions to form it. Some loss of alpha-helices is also clear. At 40 ns, one monomer attaches to the polar lipid layer, while the other continues rotating and translating until it reaches a state of apparent equilibrium, where interaction seems to occur between the C-terminal residues. In this state, both structures are nearly parallel to the lipid head layer.

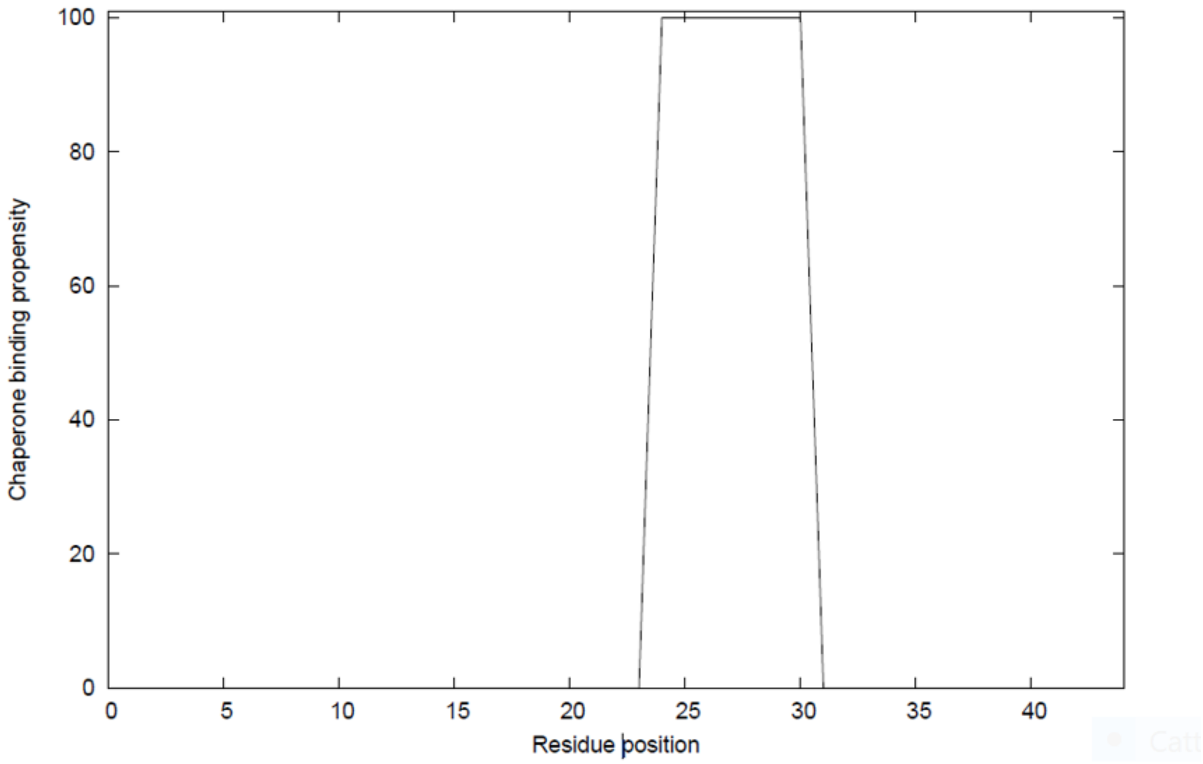

Figure S17 - The heptad sequence of ORF7b2. The graph shows a heptad sequence of ORF7b2 at positions 24-30 (MLIIFWF) as a binding site for Hsp70 (score 23.17), calculated using the specific algorithm in Limbo.switchlab (https://limbo.switchlab.org), VIB Switch Laboratory, Vrije Universiteit Brussel, Brussels, Belgium. We performed some specific tests on ORF7b2 using the Limbo-Switch-lab Server [72] for "Best Sequence", "High Specificity Prediction", and "High Sensitivity Prediction". These tests showed with a high statistical score (23.17) that ORF7b2 has, at positions 24-30, a canonical heptad sequence (MLIIFWF) specific for a potential binding to the chaperone HSP70.

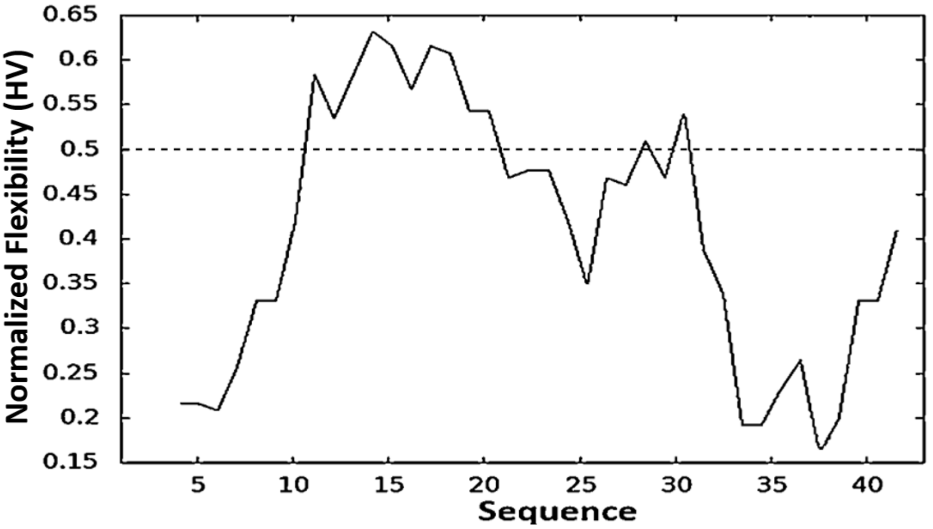

Figure S18 – Flexibility plot of ORF7b-2. The flexibility of a protein depends on the amino acid residues in the highly mobile regions. We generated the plot by calculating the hydrophobicity-volume product for consecutive quintuplets of amino acid residues [157]. The presence of small volumes and low hydrophobicity generates low average HV product values, with the minimum values representing flexibility but also disorder. The occurrence of small volumes (V) and low hydrophobicities (H) yields low mean HV product values, with minima that represent flexibility. This result agrees very well with the results of molecular dynamics, Normal Mode Analysis, and RIN analysis. We normalized the data to a 0-1 scale.
